# Modulation of Human Phenylalanine Hydroxylase by 3-Hydroxyquinolin-2(1H)-One Derivatives

**DOI:** 10.3390/biom11030462

**Published:** 2021-03-19

**Authors:** Raquel R. Lopes, Catarina S. Tomé, Roberto Russo, Roberta Paterna, João Leandro, Nuno R. Candeias, Lídia M. D. Gonçalves, Miguel Teixeira, Pedro M. F. Sousa, Rita C. Guedes, João B. Vicente, Pedro M. P. Gois, Paula Leandro

**Affiliations:** 1 Research Institute for Medicines, Faculty of Pharmacy, Universidade de Lisboa, Av. Prof. Gama Pinto, 1649-003 Lisboa, Portugal; rrlopes@ff.ulisboa.pt (R.R.L.); cstome@farm-id.pt (C.S.T.); robertorusso@fm.ul.pt (R.R.); paterna.roberta@gmail.com (R.P.); jptleandro@gmail.com (J.L.); lgoncalves@ff.ulisboa.pt (L.M.D.G.); rguedes@ff.ulisboa.pt (R.C.G.); 2 Instituto de Tecnologia Química e Biológica António Xavier, Universidade Nova de Lisboa, Av. da República, 2780-157 Oeiras, Portugal; miguel@itqb.unl.pt; 3 Instituto de Biologia Experimental e Tecnológica, Quinta do Marquês, 2780-155 Oeiras, Portugal; pedrosousa@ibet.pt; 4 Faculty of Engineering and Natural Sciences, Tampere University, Korkeakoulunkatu 8, 33101 Tampere, Finland; ncandeias@ua.pt; 5 LAQV-REQUIMTE, Department of Chemistry, University of Aveiro, 3810-193 Aveiro, Portugal

**Keywords:** protein misfolding, drug discovery, inherited metabolic disorders, protein drug interactions, pharmacological chaperones, activity chaperones

## Abstract

Phenylketonuria (PKU) is a genetic disease caused by deficient activity of human phenylalanine hydroxylase (hPAH) that, when untreated, can lead to severe psychomotor impairment. Protein misfolding is recognized as the main underlying pathogenic mechanism of PKU. Therefore, the use of stabilizers of protein structure and/or activity is an attractive therapeutic strategy for this condition. Here, we report that 3-hydroxyquinolin-2(1H)-one derivatives can act as protectors of hPAH enzyme activity. Electron paramagnetic resonance spectroscopy demonstrated that the 3-hydroxyquinolin-2(1H)-one compounds affect the coordination of the non-heme ferric center at the enzyme active-site. Moreover, surface plasmon resonance studies showed that these stabilizing compounds can be outcompeted by the natural substrate l-phenylalanine. Two of the designed compounds functionally stabilized hPAH by maintaining protein activity. This effect was observed on the recombinant purified protein and in a cellular model. Besides interacting with the catalytic iron, one of the compounds also binds to the N-terminal regulatory domain, although to a different location from the allosteric l-Phe binding site, as supported by the solution structures obtained by small-angle X-ray scattering.

## 1. Introduction

Inherited metabolic disorders (IMDs) are genetic diseases causing the impaired function of enzymes or transporters involved in intermediary metabolism. The mutational spectrum of the majority of IMDs is dominated by missense mutations (50–80%), frequently leading to protein variants with deficient catalytic activity or impaired folding. The latter results in premature protein degradation and consequently, lower cellular protein steady-state levels. Attractive therapeutic approaches for these conditions are based on the use of small molecules acting as “pharmacological chaperones” rescuing variant proteins’ folding, or as “activity chaperones” protecting enzyme activity [1,2]. Small molecules acting as pharmacological chaperones are already approved for the treatment of genetic diseases associated with protein misfolding such as Sapropterin dihydrochloride (Kuvan) from Biomarin (phenylketonuria), Tafamidis, Pfizer (specific TTR variants responsible for familial amyloid polyneuropathy), Lumacaftor, Orkambi (F508del CFTR in cystic fibrosis) and Migalastat (Galafold) from Amicus Therapeutics (responsive variant forms of α-galactosidase A causing Fabry disease) [3].

**Figure 1:**
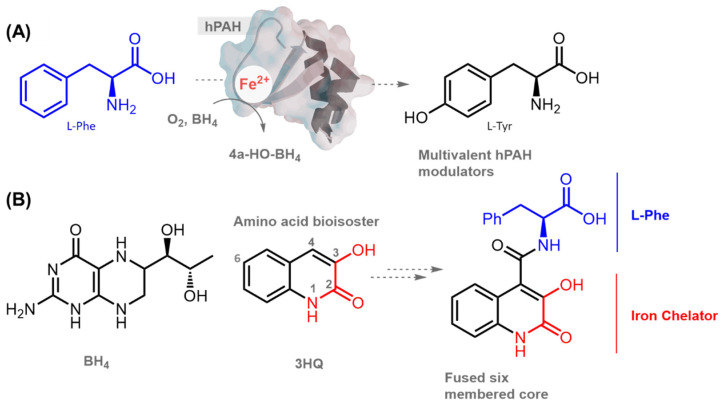
Rationale for the putative pharmacological/activity chaperone potential of 3-hydroxyquinolin-2(1H)-one (3HQ) derivatives towards human phenylalanine hydroxylase (hPAH). (**A**) Schematic representation of the reaction catalyzed by hPAH where L-phenylalanine (l-Phe) is hydroxylated to L-tyrosine (l-Tyr) in the presence of the cofactor (6R)-L-erythro-5,6,7,8-tetrahydrobiopterin (BH_4_), Fe^2+^, and oxygen. (**B**) Structural properties of the 3HQs library, displaying structural similarities with BH_4_, presence of l-Phe at the 4-position and iron-coordinative properties.

Phenylketonuria (PKU; OMIM 261600) is the most common IMD of amino acid metabolism (1:10,000 newborns) and is caused by mutations in the PAH gene, encoding phenylalanine hydroxylase (hPAH; EC 1.14.16.1). When untreated, PKU can lead to severe psychomotor developmental delay. To date, more than 1000 different mutations have been identified in PAH (BIOPKU database; http://www.biopku.org; last accessed on 13 October 2020) ≈62% being classified as missense mutations. As such, PKU is a suitable target for the development of activity chaperone-and pharmacological chaperone-based therapies. Functional hPAH assembles as a homotetramer (dimer of dimers) and harbors a non-heme iron in its catalytic site, where hydroxylation of l-phenylalanine (l-Phe) into l-tyrosine (l-Tyr) takes place in the presence of molecular oxygen and the cofactor (6R)-l-erythro-5,6,7,8-tetrahydrobiopterin (BH_4_) (Figure 1A).

Each hPAH monomer is organized into three structural domains, namely an N-terminal regulatory domain, a catalytic domain, and a C-terminal tetramerization domain. The active site where l-Phe and the BH_4_ cofactor bind is located in the catalytic domain, in a pocket composed of both hydrophobic and negatively charged residues, where a 2-His-1-carboxylate facial triad (His285, His290 and Glu330) binds to mononuclear ferric iron, which is also coordinated to three water molecules. The enzyme alternates between a so-called ‘resting’ state, where the N-terminal regulatory domain covers the active site and leads to lower substrate affinity and lower catalytic activity, and an ‘activated’ state where a rotating displacement of this domain facilitates l-Phe access to the active site leading to an enzyme with higher catalytic activity. High levels of circulating substrate stabilize the ‘activated’ state by the allosteric l-Phe binding to the interface established by the dimerization of the regulatory domains of adjacent monomers [4,5,6,7,8]. In the absence of l-Phe, BH_4_ binding has an inhibitory effect resulting from locking the protein in a non-activated state with less mobile regulatory domains, presenting increased resistance to unfolding and degradation [7,9,10]. In fact, this mechanism underlies the rationale to use the synthetic form of BH_4_ (sapropterin dihydrochloride) as a pharmacological chaperone that stabilizes some misfolded hPAH variants [11,12]. Importantly, despite BH_4_ being used in high amounts per dose (20 mg/kg body weight) and patients with more severe PKU phenotypes often being unresponsive to this molecule, the BH_4_ mode of action remains a leading strategy in the design of pharmacological chaperones to promote the enzyme stabilization and structural integrity.

Our groups previously reported an alternative strategy to design hPAH modulators based on the l-Phe structure by preparing and evaluating a series of l-Phe-iminoboronates that increased hPAH activity by a pre-activation mechanism similar to the one involving l-Phe [13]. Interestingly, to our knowledge, the catalytic iron center has never been explored to design hPAH modulators. Thus, given the ability of 3-hydroxyquinolin-2(1H)-one (3HQ) to complex metals [14], in the present study, we evaluated an in-house library of 3HQ derivatives, recently developed as anticancer agents [15]. In addition to potentially stabilizing the hPAH catalytic non-heme iron, these compounds contain a BH_4_ cofactor-like fused six membered core and offer the possibility of installing l-Phe-like motifs as substituents to improve affinity and functionality (Figure 1B). This combination of structural features of both the cofactor and the substrate along with iron coordinative functionalities should provide an excellent scaffold to promote an interaction specifically directed to the protein’s catalytic domain. We rationalized that this approach is likely to favor the discovery of compounds stabilizing the protein structure (pharmacological chaperones) and/or protecting enzyme activity (activity chaperones), that ultimately sustains cell function. These compounds should bind with enough affinity to ensure specificity without preventing l-Phe binding. Herein, we describe the biochemical and biophysical characterization of 3HQ derivatives as pharmacological/activity chaperones of hPAH, as well as their efficiency and toxicity in a cellular context.

## 2. Materials and Methods

### 2.1. Compounds Library

**Figure 2:**
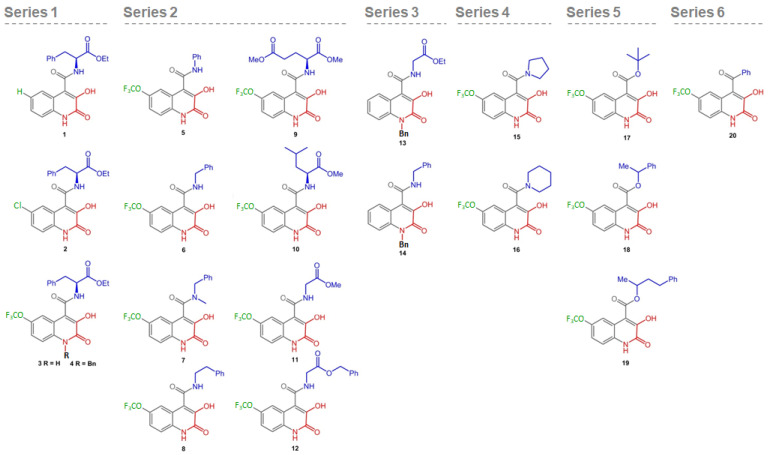
Chemical structures of 3-hydroxyquinolin-2(1H)-one (3HQ) derivatives screened. The compounds display a common 3HQ core, with variations at positions 1 (black), 4 (blue) and 6 (green); substituents include aliphatic motifs (benzyl, ethyl and methyl), aryl (phenyl) and electron withdrawing groups (chloride and trifluoromethoxy); the putative iron chelating moiety is represented in red.

The 20 3HQ derivatives (Figure 2) were synthesized according to protocols already reported by our group [15] and described in detail in the Supplementary Material for compounds 1 and 2. The synthesized 3HQ derivatives were solubilized to 10 mM in DMSO and further used at final concentrations of 50 to 200 μM in 0.5 to 2% DMSO.

### 2.2. Production and Purification of Full-Length and Truncated Recombinant hPAH

Recombinant full-length wild-type (WT) hPAH was expressed in *E. coli* Top10 cells in fusion with a hexa-histidyl peptide to allow purification by immobilized metal affinity chromatography (IMAC) using a Ni-NTA agarose (Qiagen; Hilden, Germany) essentially as described previously [16]. After IMAC purification, hPAH tetramers were isolated by size-exclusion chromatography (SEC) on a HiLoad Superdex 200 HR column (1.6 × 60 cm, GE Healthcare; Chicago, IL, USA) equilibrated with 20 mM Na-HEPES buffer containing 200 mM NaCl, pH 7.0 (SEC buffer), at a flow rate of 0.7 mL/min, at 4 °C (Supplementary Figure S1, peak 3) [13].

The truncated form of hPAH corresponding to the N-terminal regulatory domain (hPAH-RD^1–120^) was expressed in E. coli TB1 cells in fusion with maltose binding protein (MBP) and a sequence recognized by Factor Xa (FXa) (MBP-FXa-hPAH-RD^1–120^) to allow further removal of the MBP tag, as described previously [17] (Supplementary Figure S2, peak 2). The MBP-FXa-hPAH-RD^1–120^ protein was purified by affinity chromatography using an amylose resin (New England Biolabs; Ipswich, MA, USA). SEC was employed to isolate the hPAH-RD^1–120^ dimers as described above. The protein concentrations were determined by the Bradford method, using bovine serum albumin as the standard.

### 2.3. Enzymatic Activity Assays

The hPAH activity was measured essentially as previously described [13]. Briefly, the enzymatic reaction was performed in a 200 μL final volume, containing 250 mM Na-HEPES, pH 7, 0.1 mg/mL catalase (Sigma-Aldrich; St. Louis, MO, USA), 5 μg (final concentration of 0.112 μM of tetramer) of recombinant hPAH tetramers and 100 μM (NH_4_)_2_Fe(II)SO_4_. Using 100 μM l-Phe and 100 μM of tested compound, three experimental conditions were used to study the compounds’ modulatory effect, namely: non activated (NA), 3HQ-activated (3HQ-A) and substrate/3HQ-activated (Phe/3HQ-A) [13] (Supplementary Figure S3). The reaction was started by addition of 75 μM BH_4_ (Schircks Laboratories; Bauma, Switzerland) prepared in 5 mM ascorbic acid. In the NA assay, the l-Phe substrate and tested compounds were added together with 75 µM BH_4_ at the start of the hydroxylation reaction. In the 3HQ-A condition, hPAH was pre-incubated with the tested compound for 4 min at 25 °C and the reaction was started by the addition of BH_4_ together with l-Phe. In the Phe/3HQ-A condition, hPAH was pre-incubated simultaneously with l-Phe and the tested compound (4 min, 25 °C). Appropriate control reactions were performed where the compounds were omitted and 1% DMSO was used. Kinetic parameters were determined using variable concentrations of l-Phe (25 to 2500 μM) and fixed concentrations of BH_4_ (75 μM) and tested compound (100 μM). After a 1-min incubation, the reaction was stopped, and the amount of l-Tyr produced was quantified by HPLC as previously described [13]. The specific hPAH enzymatic activity is expressed as nmol of l-Tyr produced during 1 min per mg of protein (nmol l-Tyr.min^−1^.mg^−1^). The steady-state kinetic data were analyzed by nonlinear regression analysis using GraphPad Prism (La Jolla, CA, USA). Data was fitted to the modified Hill equation accounting for cooperative substrate binding as well as substrate inhibition [18] (Equation (1)), to the modified Michaelis–Menten equation which accounts for substrate inhibition (Equation (2)) and to the non-modified Michaelis–Menten equation:

*v* = (*V*_max_ + *V*_i_ ([*S*]^x^/*K*_i_^x^))/(1 + (*K*^h^/[*S*]^h^) + ([*S*]^x^/*K*_i_^x^)) (1)

*v* = (*V*_max_ [*S*])/(*K*_m_ + [*S*](1 + [*S*]/*K*_i_)) (2)

The hPAH time-dependent loss of activity at a fixed temperature was assessed in a period of 120 min, in the absence and presence of compounds. In these assays, the enzymatic reaction was performed essentially as described above for the Phe/3HQ-A condition. Compounds (100 μM) were pre-incubated with the enzyme at 42 °C. After a 30, 45, 60, 90 and 120 min period, an aliquot of the protein (5 μg) was incubated, at 37 °C, with 100 μM l-Phe for 4 min, and the reaction proceeded as above at 37 °C [19].

For the above-described activity assays, three independent assays were always performed.

### 2.4. Differential Scanning Fluorimetry

Thermal denaturation profiles of recombinant hPAH were obtained by differential scanning fluorimetry (DSF) in a C1000 Touch thermal cycler equipped with a CFX96 optical reaction module (Bio-Rad; Hercules, CA, USA) as previously described [13]. Briefly, DSF assays (*n* = 3) were performed using 100 μg/mL hPAH tetramers (final concentration of 0.45 μM of tetramer), SYPRO Orange (5000-fold commercially available stock solution; Invitrogen; Carlsbad, CA, USA) at a 2.5-x final concentration and ramping the temperature from 20 to 90 °C, at 1 °C/min. Fluorescence data were acquired using the FRET channel. Data were processed using CFX Manager Software V3.0 (Bio-Rad) and the GraphPad Prism. Temperature scan curves were fitted to a biphasic dose–response function and the *T*_m_ values were obtained from the midpoint of the first (*T*_m1_) and second transitions (*T*_m2_) corresponding, respectively, to the thermal denaturation of the regulatory and catalytic domains [20,21]. The *T*_m_ values in the presence of 3HQ were compared to those obtained in the presence of 1% DMSO (vehicle control) and a change in the *T*_m_ was considered significant when the |Δ*T*_m_| ≥ 2 °C [22].

### 2.5. Limited Proteolysis by Trypsin

Limited proteolysis of purified hPAH was performed as previously described [23], at 25 °C in SEC buffer and with a trypsin (Sigma-Aldrich) to hPAH ratio of 1:200 (by mass; final hPAH protein concentration 300 μg/mL), in the presence of 100 µM of 3HQ derivatives or 1% DMSO. Control assays were also performed in the presence of 100 μM and 1 mM l-Phe. At different time points (0 to 60 min), aliquots of the reaction were mixed with soybean trypsin inhibitor (Sigma-Aldrich) at a protease to inhibitor ratio of 1:1.5 (by mass) and analyzed by SDS-PAGE (10% polyacrylamide). The band corresponding to full-length hPAH was quantified by densitometric analysis using the Quantity One 1D software v4.6.3 (Bio-Rad). Data were fitted to a single exponential decay equation to obtain the decay constant of proteolysis (*k*_P_). Assays were performed in duplicate and data represent the mean ± SEM.

To rule out compounds that inhibit/inactivate trypsin, changes in the cleavage rate of N-(Benzyloxy)carbonyl-glycylglycylarginyl-7-amido-4-methylcoumarin (Z–Gly–Gly–Arg–AMC; Bachem, Bubendorf, Switzerland), a synthetic substrate of trypsin, were monitored by fluorimetric measurements in a microplate reader (FLUOstar Omega; BMGLabtech, Ortenberg, Germany). The assays (*n* = 3) were performed in a 96-well plate, in SEC buffer and in a final volume of 200 μL containing 1.5 μg/mL of trypsin, 50 μM of Z–Gly–Gly–Arg–AMC and 100 μM of 3HQs derivatives. Fluorescence was measured in real-time, with λ_exc_ 360 nm and λ_em_ 460 nm, during 30 min at 25 °C. Controls were performed using substrate alone with 1% DMSO (negative control) and trypsin with no compounds and 1% DMSO (positive control).

### 2.6. Electron Paramagnetic Resonance Spectroscopy

EPR spectra of hPAH (at ≈100 μM monomer) in the absence or presence of equimolar amounts of 3HQs were recorded at 4K in a Bruker EMX spectrometer (Billerica, MA, USA) equipped with an ESR-900 continuous flow helium cryostat from Oxford Instruments (Abingdon, Johnson, UK). Spectra were recorded for control samples containing 100 μM FeCl_3_ solutions incubated with equimolar amounts of 3HQs to evaluate direct binding of the compounds to free Fe^3+^ in solution. Microwave frequency: 9.39 GHz; microwave power, 2 mW; modulation amplitude, 1 mT.

### 2.7. Surface Plasmon Resonance

The affinity of hPAH for 3HQs was evaluated by surface plasmon resonance (SPR) using a Biacore 4000 (GE Healthcare) instrument. All assays were carried out at 25 °C. Optimization of hPAH immobilization on a carboxymethylated dextran (CM5) matrix of the sensor chip was initiated by performing a pH scouting, using as buffers 10 mM sodium acetate (pH 5.0, 5.2, 5.5 and 5.8), 10 mM Bis-Tris (pH 6.0, 6.2 and 6.5), and 10 mM sodium phosphate (pH 7.0). hPAH was diluted to 10 µg/mL in the best immobilization buffer (10 mM sodium acetate pH 5.5) and immobilized onto the CM5 sensor chip using the standard amine coupling procedure. The HBS-N buffer, consisting of 10 mM HEPES pH 7.4 and 150 mM NaCl, was used as background buffer. Prior to immobilization, the carboxymethylated surface of the chip was activated with a 1:1 ratio of 400 mM 1-ethyl-3-(3-dimethylaminopropyl)-carbodiimide and 100 mM N-hydroxysuccinimide for 10 min. The hPAH was coupled to the surface with a 1 to 2 min injection time at a flow rate of 10 μL/min in order to reach 2000 to 5000 RU. The remaining activated carboxymethylated groups were blocked with a 7 min injection of 1 M ethanolamine pH 8.5.

The 3HQs were directly diluted in running buffer (10 mM HEPES, 150 mM NaCl, 5 mM MgCl_2_, 0.1 mM EDTA, 0.05% (*v*/*v*) Tween-20, 1 mM DTT, pH 7.2, and 2% DMSO) and injected at ten different concentrations using two-fold dilutions series, from 200 µM to ≈ 0.4 μM. The interaction between hPAH and l-Phe in the ≈ 2–1000 μM range was also analyzed, for comparison with literature values [24,25]. Moreover, l-Phe (at 1 mM) was used as a positive control to collect information on surface integrity and activity throughout the experiment. For competition experiments, 1 mM l-Phe was added to the running buffer. All sensorgrams were processed by first subtracting the binding response recorded from the control surface (reference spot), followed by subtracting an average of the buffer blank injections from the reaction spot. Interactions were assessed from the obtained plots of steady-state SPR response levels against 3HQs concentration, employing the provided Biacore 4000 evaluation software.

### 2.8. Light Scattering Assay

Self-assembly of the dimeric forms of hPAH-RD^1–120^ was followed in real-time at 25 °C upon cleavage of MBP-FXa-hPAH-RD^1–120^ with FXa (New England Biolabs) to release the MBP fusion partner. Assays (*n* = 3) were performed in the absence or presence of tested compounds as described in [17]. Briefly, FXa (at a final ratio 1:150 (*w*/*w*) relative to the fusion protein) was added to the reaction mixture containing the MBP-FXa-hPAH-RD^1–120^ fusion protein (0.74 mg/mL), 20 mM Na-HEPES, 100 mM NaCl, pH 7.0 and tested 3HQ (100 μM). The increase in absorbance at 350 nm was followed for 180 min on a FLUOstar Omega (BMG Labtech) microplate reader. A shaking step was introduced before each measurement. Appropriate control assays were performed and included: (i) a positive control consisting of MBP-FXa-hPAH-RD^1–120^ in the presence of FXa, 1% DMSO and in the absence of compounds; and (ii) a negative control consisting of MBP-FXa-hPAH-RD^1–120^ in the absence of FXa and compounds. For all the assays, aliquots of the aggregation reaction were collected at the end of the reaction (180 min) and analyzed by SDS-PAGE to confirm the extent of MBP cleavage by FXa. In addition, the effect of tested compounds on FXa activity was monitored using the synthetic FXa substrate Boc–Ile–Glu-Gly–Arg–AMC (t-(Butyloxycarbonyl)-isoleucylglutamylglycylarginyl-7-amido-4-methylcoumarin; from Bachem). Assays (*n* = 3) were performed in a 96-well plate, in a final volume of 200 μL containing 20 mM Na-HEPES, 100 mM NaCl, pH 7.0, 5 μg/mL of FXa, 50 μM of Boc–Ile-Glu–Gly–Arg–AMC and 100 μM of 3HQ derivatives. Fluorescence was measured in real-time (λ_exc_ 360 nm and λ_em_ 460 nm) during 30 min, at 25 °C. Controls were performed using substrate alone with 1% DMSO (negative control) and FXa with no compounds and 1% DMSO (positive control).

### 2.9. Studies in Eukaryotic Cells

HEK-293T cells (ATCC CRL-11268) were grown in RPMI 1640 medium (Gibco Life Technologies¸ Carlsbad, CA, USA) and supplemented with heat inactivated (60 °C for 30 min) fetal bovine serum (10%; *v*/*v*), penicillin (100 U/mL) and streptomycin (0.1 mg/mL) at 37 °C, in 5% CO_2_ in a humidified atmosphere. 1 × 10^5^ cells were transfected with 750 ng of DNA (eukaryotic vector expressing hPAH; pEF-DEST51-PAH-WT) using lipofectamine (Invitrogen) as the transfection agent. Compounds (50 and 100 μM) or DMSO (0.5 and 1%) were added 4 h after transfection, and cells were further incubated for 24 h. For sample collection cells were trypsinized with 500 μL of TripLE Express (Gibco Life Technologies), centrifuged at 200× *g* for 5 min, at 4 °C, and washed with PBS. The pellet was resuspended in 50 μL of PBS with 200 μM PMSF and cells were disrupted by passage (≈20×) through a fine needle. The cell debris were removed by centrifugation at 14,000× *g* for 5 min at 4 °C and the obtained cell lysates were used for enzymatic activity assays and western blot analysis. Three independent cell cultures were performed.

For the stability assays, after 24 h incubation with compounds, protein translation was stopped by the addition of 10 μg/mL puromycin (Sigma-Aldrich). Cells were collected at 0, 1, 3, and 6 h after blockage of translation. Two independent cell cultures were performed.

The hPAH activity of cell lysates was measured essentially as described in [26] in a final volume of 200 μL containing 1 mM l-Phe, 250 mM Na-Hepes, pH 7, 0.1 mg/mL catalase and 20 μL of cell lysate. After 4 min of pre-incubation, 100 μM (NH_4_)_2_Fe(II)SO_4_ was added to the mixture. Two min after ferrous ammonium sulphate addition, the reaction was initiated by the injection of BH_4_ (75 μM). Tyrosine production was assessed by measuring the increase in fluorescence intensity (λ_exc_ 274 nm and λ_em_ 304 nm) in a FLUOstar Omega microplate reader (BMG Labtech), at 25 °C, during 30 min. Enzyme activity was determined as the amount of l-Tyr produced per min per mg of total protein. For each cell culture enzymatic assays were performed in triplicate.

For western blot analysis of cell lysates, 4 μg of total protein were applied on an 8 to 16% mini-protean TGX precast polyacrylamide gel (Bio-Rad), and the proteins were transferred to a polyvinylidene fluoride (PVDF; GE Healthcare) membrane and visualized by immunodetection using mouse anti-PAH (MAB 5278; Millipore; Burlington, MA, USA) and mouse anti-glyceraldehyde 3-phosphate dehydrogenase (GAPDH (loading control); sc-365062, Santa Cruz Biotechnology; Dallas, TX, USA) as the primary antibodies and the anti-mouse IgG-HRP (W402B, Promega; Madison, WI, USA) as the secondary antibody. Immunoblots were developed with enhanced chemiluminescent agents (ECL Prime; GE Healthcare) and the images were acquired in a charge-coupled device imager ChemiDoc XRS (Bio-Rad) using the Quantity One software (Bio-Rad).

Cytotoxicity of selected 3HQs was assessed in HEK-293T cells using the Alamar Blue assay as a general cell viability endpoint method that monitors the cellular metabolic state, and the propidium iodide (PI) exclusion assay was used to evaluate membrane integrity essentially as described [27,28]. The 3HQs were tested at a final concentration of 50 and 100 μM (two independent assays; five replicates each). Control assays were performed using 1% DMSO (negative control), or SDS at 1 mg/mL as a positive control of cell death in the PI assay. HEK-293T cells were seeded in sterile flat bottom 96-well tissue culture plates, in RPMI 1640 culture medium, supplemented with 10% fetal bovine serum, 100 units/mL of penicillin G (sodium salt), 100 μg/mL of streptomycin sulphate and 2 mM L-glutamine, at a cell density of 2 × 10^4^ cells per well. Cells were incubated at 37 °C and 5% CO_2_. The culture medium was replaced by fresh medium (supplemented as above) containing the different 3HQs to be analyzed. After 24 h incubation, the culture medium was replaced by 0.3 mM PI (Sigma-Aldrich) in fresh medium (stock solution 1.5 mM in DMSO) and fluorescence was measured (λ_exc_ 485 nm, λ_em_ 590 nm) in a FLUOstar Omega microplate reader. The Alamar Blue assay was performed by replacing the culture medium with fresh medium containing 5 mM resazurin (Invitrogen). The cells were further incubated for 3 h and fluorescence was measured (λ_exc_ 530 nm, λ_em_ 590 nm) in a FLUOstar Omega microplate reader (BMG Labtech). The relative PI uptake and cell viability (%) were calculated using Equations (3) and (4), respectively.

Relative PI uptake = Fluorescence intensity_sample_/Fluorescence intensity_negative control_ (3)

Relative cell viability (%) = Fluorescence intensity_sample_/Fluorescence intensity_control_ × 100 (4)

### 2.10. Molecular Modelling Studies

To study and rationalize the influence of different 3-hydroxyquinolin-2(1H)-one substituents on hPAH molecular recognition, compounds **3**, **8**, **9**, and **11** were subjected to molecular docking simulations. These compounds were built and protonated at pH 7.4, and geometry was optimized using the Molecular Operating Environment (MOE) software, version 2019.01 suite (Chemical Computing Group Inc; http://www.chemcomp.com (accessed on 30 December 2019); Montreal, QC, Canada). All molecules were submitted to a conformational search and energy minimization as implemented in MOE 2019.1. Conformers were created by a systematic search generating rotatable bond angle combinations. Each dihedral angle combination was subject to an energy minimization by means of the Amber12: EHT force field. The global minimum of each molecule was submitted to docking simulations. For the docking simulation studies, the target models of hPAH were designed starting from Protein Data Bank (PDB) crystallographic structures 1MMT and 3PAH. The original PDB entries were selected taking into account the source organism (*Homo sapiens*), the best available X-ray resolution and the co-crystallized ligands. Before being used in the docking simulations, all the structures were submitted to the protein preparation tool of the MOE 2019.01 software package, to remove the co-crystallized inhibitors, add hydrogen atoms at pH 7.4, assign the correct protonation and tautomeric states, missing residues, and to remove all crystallographic water molecules.

All the docking simulations (non-covalent) were performed using GOLD 5.2 program (CCDC; Cambridge, UK) [29]. The hPAH binding site was defined at the iron center in the protein catalytic site, and the binding site radius was set to 20 Å. No constraints were used in any calculation. Flexible ligand sampling was considered in the docking procedure. All other parameters were set to defaults for the GOLD docking process. Molecular docking studies were then performed using the GoldScore scoring function from GOLD 5.2 software package and each ligand was subjected to 1000 docking runs. The docking protocol was validated for all the four structures prepared re-docking the crystallographic ligands and their poses were reproducible with root-mean-square deviations (RMSD’s) below 1.5 Å.

### 2.11. Small-Angle X-ray Scattering (SAXS)

Small-angle X-ray scattering experiments were performed at the Diamond Light Source B21 [30] beamline at a wavelength of 0.95 Å. Tetrameric hPAH was thawed on ice, centrifuged at 12,000× *g* for 8 min at 4 °C, and quantitated using a NanoDrop (molar extinction coefficient: 10.09 mg/mL/cm) prior to data acquisition. Measurements were performed in static mode with the automated sample changer at 20 °C using a protein concentration of 86 μM (monomer) in 20 mM Na-HEPES, 200 mM NaCl, pH 7.0 in the absence or presence of 2% DMSO. l-Phe and compound 9 were tested at a final concentration of 200 μM in 2% DMSO. Data were recorded using a Pilatus 2M detector covering a momentum transfer 0.0031 < s < 0.380 Å-1 (s = 4π sin θ/λ, where 2θ is the scattering angle). The data were processed and analyzed using the ATSAS program suite [31]. The sample frames were monitored for radiation damage, the selected frames were averaged, and the buffer contribution was subtracted using PRIMUS [32]. The radius of gyration (*R*_g_) and the maximum particle dimension (*D*_max_) were estimated from the Guinier approximation using PRIMUS and from the pair-distribution function *P(r)* using the GNOM package [33], respectively. Fitting of the experimental curves and the theoretical curves computed from SASBDB models was performed using CRYSOL [31].

## 3. Results

### 3.1. Effect of 3HQ Derivatives on hPAH Activity and Thermal Stability

**Figure 3:**
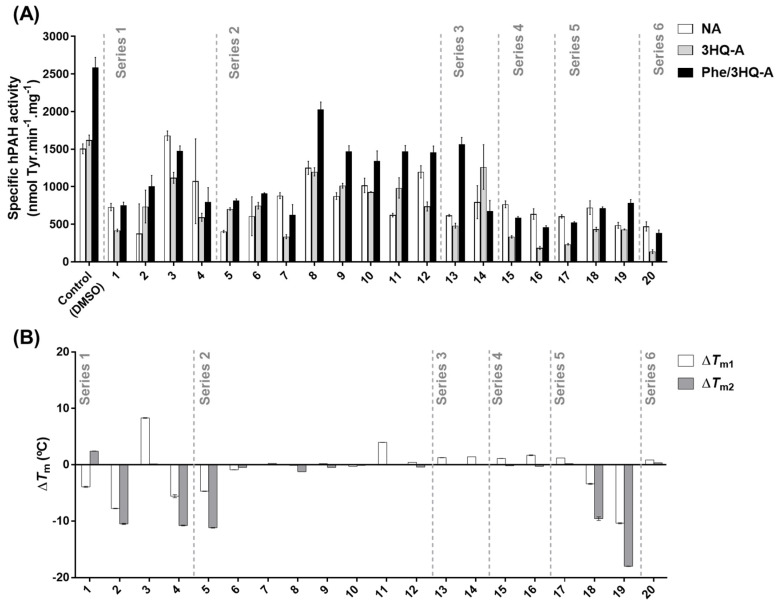
Effect of the 3-hydroxyquinolin-2(1H)-one (3HQ) derivatives library on the specific enzyme activity and thermal stability of human phenylalanine hydroxylase (hPAH). (**A**) Catalytic activity was determined in three different conditions: Non-activated (NA) where the substrate and tested 3HQs were added together with the cofactor at the start of the enzymatic reaction without pre-activation; 3HQ-activated (3HQ-A), where the enzyme was pre-incubated with the tested compound for 4 min at 25 °C and the reaction was started by the simultaneous addition of the substrate and cofactor; 3HQ/Substrate-activated (Phe/3HQ-A), where the enzyme was pre-incubated simultaneously with the substrate and the tested compound (4 min, 25 °C). (**B**) The mid-point of thermal denaturation (*T*_m_) for the regulatory (*T*_m1_) and catalytic (*T*_m2_) hPAH domains were determined by differential scanning fluorimetry and the values were compared to the control to calculate Δ*T*_m_. In (**A**,**B**) 3HQs were tested at 100 μM in 1% DMSO (V) and data were compared to the control assays (1% DMSO and absence of 3HQ); data represent the mean ± SD from three independent assays.

The 3HQ in-house library comprised 20 compounds with different substituents at positions 1, 4 and 6 (Figure 2). The substituents in position 4 include l-Phe-like moieties (compounds **5–8**, **12**, **14** and **18–19**) predicted to enhance selectivity towards the catalytic site, while different electron withdrawing substituents at position 6 are expected to increase the acidity of the hydroxy group and thus the coordination with the iron center. The 3HQ derivatives were first screened by evaluating their modulatory effect on hPAH activity and thermostability (Figure 3).

To assess the compounds’ effect on both catalysis and enzyme pre-activation by the substrate, the catalytic activity was monitored in three assays under different conditions, namely NA, 3HQ-A, and Phe/3HQ-A (Supplementary Figure S3). Obtained data were compared to the corresponding control assays where only 1% DMSO was used (Figure 3A; control). In the absence of compounds (control reaction; 1% DMSO) recombinant hPAH non pre-activated by l-Phe (‘resting state’; NA control) presented an enzymatic activity of 1504 ± 66 nmol Tyr.min^−1^.mg^−1^. Pre-incubation with 1% DMSO (3HQ-A control) did not increase enzyme activity (1620 ± 67 nmol Tyr.min^−1^.mg^−1^; *p* = 0.1), whereas incubation with 100 μM l-Phe (substrate-activated; Phe/3HQ-A control) resulted in a 1.7-fold higher activity (2590 ± 130 nmol Tyr.min^−1^.mg^−1^). When the data of the three compound assay conditions were compared with the respective control (Figure 3A), with the exception of compound **3** (Series 1) where a 1.1-fold increase (*p* < 0.0001) was obtained for the NA condition, an inhibition of enzyme activity was observed, although to different extents. Among the compound series tested, and when compared to the control assays, series 4, 5, and 6 promoted the highest decrease in enzyme activity in all the tested conditions (NA < 50%, 3HQ-A < 27% and Phe/3HQ-A < 30% residual activity). Compounds from Series 3 showed different effects. In particular, compound **14** did not allow substrate activation (Phe/3HQ-A vs. 3HQ-A) and pre-incubation with compound **13** decreased enzyme activity (3HQ-A vs. NA). Compounds **1**, **2**, and **4** (Series 1) and compounds **5** to **7** (Series 2) also inflicted high decreases in enzyme activity (compared to the respective control assays).

According to the above results, six molecules—compounds **3** (Series 1) and **8** to **12** (Series 2)—stand out among the tested 3HQ derivatives for their effect on hPAH activity. Compound **3** (Series 1), containing an l-Phe motif as the 4-substituent, is the only 3HQ derivative that fosters an increased hPAH activity in the NA condition. In addition, it revealed a low impact on the activity in the 3HQ-A (69% activity) and Phe/3HQ-A conditions (57% activity) when compared to the respective control. Moreover, it still allowed a 1.3-fold activation by l-Phe (Phe/3HQ-A vs. 3HQ-A condition). From series 2, compound **8**, featuring a 2-phenylethanamide substituent that mimics the l-Phe side chain, retained 74 to 83% activity under all three conditions and promoted 1.7-fold enzyme activation (Phe/3HQ-A vs. 3HQ-A); **9** and **10**, despite affecting hPAH activity in the NA condition (58%, and 68% activity, respectively) afforded both 1.4-fold activation by l-Phe, with compound **10** slightly inhibiting hPAH activity in the 3HQ-A condition (0.9-fold); compound **11** was able to enhance the enzyme activity 1.6-fold (3HQ-A vs. NA), and afforded 1.5-fold activation by l-Phe (Phe/3HQ-A vs. 3HQ-A); compound **12**, although allowing substrate activation (Phe/3HQ-A vs. C-A) it did not promoted enzyme activation (0.6-fold decrease; 3HQ-A vs. NA).

Regarding protein thermostability, upon thermal denaturation at a linear temperature gradient, hPAH displays a two-phase transition characterized by two melting temperatures (*T*_m_) attributed to the denaturation of the regulatory (*T*_m1_) and the catalytic (*T*_m2_) domains [20]. The *T*_m_ values for the DMSO-incubated control sample were *T*_m1_ = 43.4 ± 0.6 °C and *T*_m2_ = 53.5 ± 0.4 °C, which increased in the presence of 1 mM l-Phe to 51.0 ± 0.2 °C and 58.4 ± 0.04 °C, respectively (Supplementary Figure S4). The vast majority of the tested 3HQ derivatives did not contribute to stabilization or destabilization (|Δ*T*_m_| ≥ 2 °C) of the regulatory and catalytic domains (Figure 3B and Supplementary Table S1). However, the hPAH regulatory domain became stabilized upon incubation with compounds **3** (Δ*T*_m1_ = +8.3 ± 0.06 °C) and **11** (Δ*T*_m1_ = +4.0 ± 0.01 °C). Five compounds (**2**, **4**, **5**, **18**, and **19**) destabilized the regulatory and catalytic domains, leading to Δ*T*_m_ ranging from −3.41 ± 0.09 (compound **18**; *T*_m1_) and −17.99 ± 0.03 °C (compound **19**; *T*_m2_).

Combining the observed effect on the activity and thermal stability of hPAH, induced by the 20 screened 3HQ derivatives, and having as a pre-requisite that compounds should not decrease the protein thermal stability and should contribute to enzyme activation (higher 3HQ-A vs. NA) allowing further activation by the substrate (Phe/3HQ-A vs. 3HQ-A), compounds **3**, **8**, **9**, and **11** were selected for a detailed characterization as they were the most promising candidate molecules.

### 3.2. Effect of Selected 3HQ Derivatives on the Kinetic Parameters of hPAH

**Figure 4:**
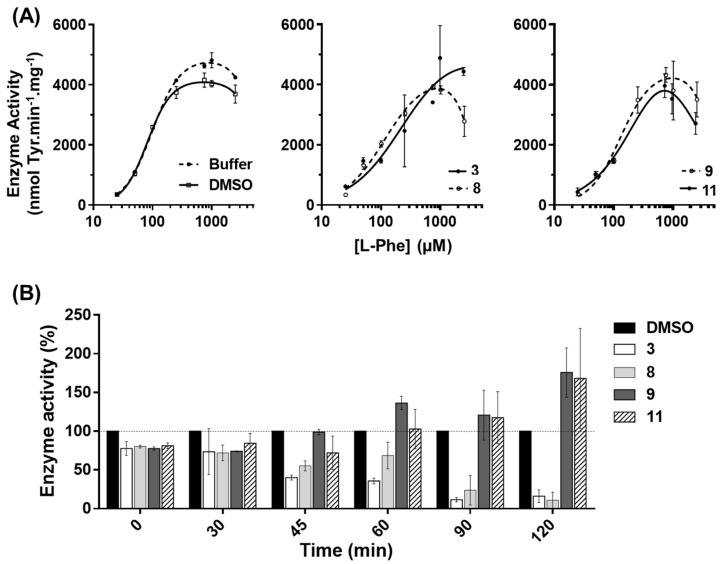
Effect of selected 3HQ derivatives on human phenylalanine hydroxylase (hPAH) catalytic response to increasing concentrations of l-Phe and enzyme activity resistance to thermal inactivation. (**A**) The hPAH activity was assayed at standard conditions (25 to 2500 μM l-Phe, 75 μM BH_4_ and 25 °C) in the presence of reaction buffer, 1% DMSO and 100 μM of compounds **3**, **8**, **9**, and **11**; data represent mean ± SD of three assays; data were fitted to the modified equations of Hill [18] (Equation (1)) or Michaelis–Menten (Equation (2)) accounting for substrate inhibition or the non-modified Michaelis–Menten equation. (**B**) Time-dependent thermal inactivation profile of hPAH in the absence and presence of selected 3HQ derivatives; residual enzymatic activity was determined after 30, 45, 60, 90, and 120 min pre-incubation at 42 °C in the presence of 1% DMSO (control) and 100 μM of selected compounds; the enzyme activity obtained for compounds **3**, **8**, **9**, and **11** were compared with the activity obtained for the control sample (1% DMSO) for each incubation time, which was considered 100%. Data represent the mean ± SD from three independent assays.

The effect of compounds **3**, **8**, **9**, and **11** on the hPAH steady-state kinetics was studied over a substrate range of 25 μM to 2.5 mM l-Phe. The obtained hPAH kinetic parameters (*V*_max_, *S*_0.5_, *h* and catalytic efficiency) for the control sample in the presence of 1% DMSO were in the range of those described in the literature [8,34] (Table 1, Reaction buffer), including the reported substrate inhibition (Figure 4A).

In the presence of compounds **3**, **8**, **9**, and **11**, inhibition of enzyme activity at high l-Phe concentrations was maintained for compounds **8**, **9**, and **11** (Figure 4A). While for **8** and **9** the data best fitted to the modified Hill equation of LiCata and Allewel [18] (cooperative substrate binding and substrate inhibition; Equation (1)), for **3** and **11**, a loss of allostery was observed, and for compound **11**, the data best fitted to the modified equation of Michaelis–Menten, which accounts for substrate inhibition (Equation (2)). Although compound **9** did not change *V*_max_ and *S*^0.5^ significantly, it resulted in lower catalytic efficiency. In the presence of compounds **3** and **8**, the higher *K*_m_ or *S*_0.5_ for l-Phe and the unchanged *V*_max_ suggest that these compounds are weak competitive inhibitors. Compound **11**, which led to the lowest catalytic efficiency, increased both the *V*_max_ and the *K*_m_. Compounds **3** and **11**, which exhibited higher *K*_m_ for l-Phe, also lead to a loss of cooperativity.

### 3.3. Affinity of Selected 3HQs for hPAH

**Figure 5:**
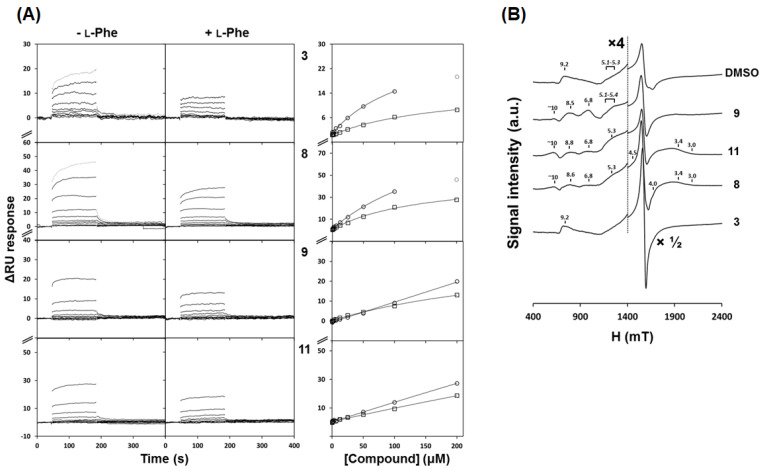
Effect of selected 3HQ derivatives on human phenylalanine hydroxylase (hPAH) properties (affinity for the substrate and electron paramagnetic resonance (EPR) spectra). (**A**) Affinity of hPAH for selected 3HQ derivatives analyzed by surface plasmon resonance (SPR); left panel shows the sensorgrams for the binding of compounds **3**, **8**, **9**, and **11**, at concentrations from 0.4 to 200 µM, in the absence (-l-Phe) and presence of 1 mM l-Phe (+l-Phe) in the running buffer; the right panel shows the effect of compound concentration on the apparent equilibrium binding to hPAH tetramers in the absence (○) and presence of 1 mM l-Phe (☐) in the running buffer. (**B**) EPR spectra, of hPAH in the absence and presence of compounds; hPAH (at ≈100 μM monomer) was mixed with equimolar amounts of 3HQs (**3**, **8**, **9**, and **11**) and spectra were recorded at 4K in a Bruker EMX spectrometer equipped with an Oxford Instruments ESR-900 continuous flow helium cryostat; microwave frequency: 9.39 GHz; microwave power, 2 mW; modulation amplitude, 1 mT; DMSO was used at 1% concentration.

To assess the potential of the selected 3HQs as activity/pharmacological chaperones for hPAH, the relative affinity was assessed by surface plasmon resonance (SPR). In line with its predicted pI of 6.1, hPAH was best immobilized on the CM5 chip in sodium acetate buffer at pH 5.5, similarly to previously reported SPR studies on hPAH [24,25]. Compound binding was evaluated at 10 different concentrations up to 200 μM, a common scouting concentration for screening routines, and l-Phe binding assay was used as a positive control. Under the tested conditions, l-Phe binding to hPAH occurred with a steady-state affinity constant (*K*_D_) of 47 ± 8 μM, in accordance with previously reported SPR-determined values (97 ± 6 μM and 136 ± 2 μM) [24,25]. As for the selected 3HQs, a transient binding behavior, characterized by fast association and dissociation rate constants, was observed on the interactions with the immobilized hPAH (Figure 5A, left panel). The interaction profiles of compounds **3**, **8**, **9**, and **11** resulted in sensorgrams with a good correlation between the calculated surface activities and the observed response units (Figure 5A, left panel). The steady-state response units were analyzed as a function of compound concentration (Figure 5A, right panel) to estimate the *K*_D_ of hPAH interaction with each compound. As observed in Figure 5A (right panel), despite none of the compounds affording a steady-state response unit saturation up to the maximal concentration herein employed (200 μM), a lower limit of *K*_D_ values could be estimated for compounds **3** and **8** (*K*_D_ ≥ 121 and ≥ 200 μM, respectively). For compounds **9** and **11**, the steady-state response units increased linearly with compound concentration up to 200 μM (Figure 5A, right panel), hinting for at least mM affinity. Importantly, in the presence of saturating concentrations of the substrate l-Phe, injection of the 3HQ derivatives resulted in a decrease of the variation of the response units (ΔRU) (Figure 5A) when compared to the values obtained for the individual analysis of ligand binding (l-Phe or 3HQs) on identical surface densities. This inhibitory effect of l-Phe is particularly significant on the interaction between compound **3** and hPAH (>50% ΔRU reduction).

### 3.4. Interaction between 3HQs and hPAH Iron Centre Monitored by Electron Paramagnetic Resonance (EPR) Spectroscopy

The presence of a putative iron binding moiety in the structure of the studied 3HQ derivatives lead us to employ EPR spectroscopy to monitor changes in the environment of the non-heme mononuclear iron active site metal center as a consequence of ligand binding. When isolated, hPAH has an EPR spectrum dominated by a characteristic signal centered at g = 4.25 (with an E/D value of ≈ 0.33) attributed to the high-spin (*S* = 5/2) mononuclear oxidized ferric center (Figure 5B), in line with those reported [35]. In addition, weak broad resonances were observed, centered at g ≈ 9.2 and at g ≈ 5.1 to 5.3. Incubation with the selected 3HQs induced spectral changes that indicate direct interaction with the catalytic Fe^3+^ center (Figure 5B). In order to evaluate whether the observed effects can be correlated with an interaction between the 3HQs and free iron, control spectra were recorded of FeCl_3_ incubated with the selected 3HQs (Supplementary Figure S5), which did not disclose any obvious effects of 3HQs on free iron. As for hPAH, compound **3** resulted in a significant increase in the intensity of this main band at g = 4.25, maintaining the minor broad resonances, similar to the effect observed upon incubation of hPAH with its substrate l-Phe [35]. Upon incubation of hPAH with compounds **8**, **9**, or **11**, while the main signal at g = 4.25 remained essentially unaffected, less rhombic EPR signals appeared with g values ranging from ≈ 3.1 to ≈ 10, with E/D values from 0.04 to 0.17 assigned to the different Kramer’s doublets (Supplementary Table S2).

### 3.5. Response of hPAH to Limited Proteolysis by Trypsin in the Presence of Selected 3HQ Derivatives

**Figure 6:**
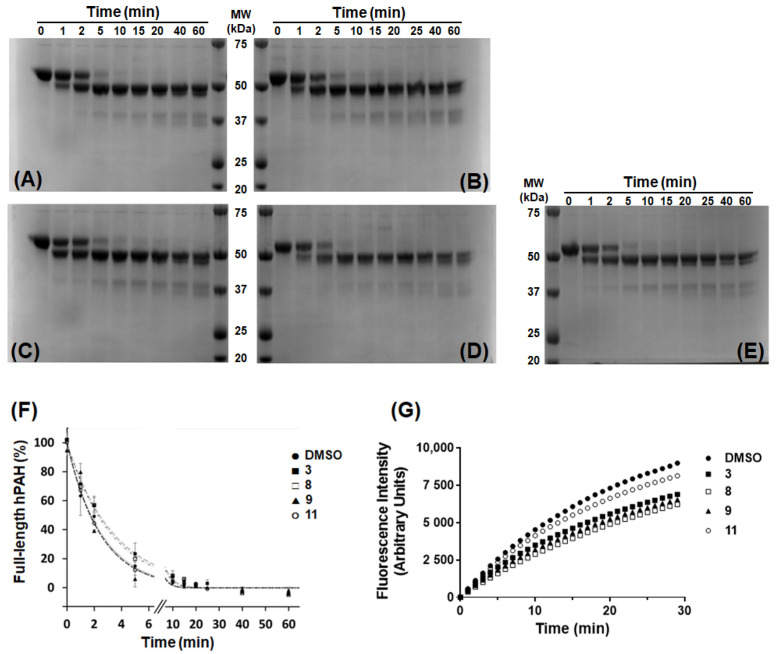
Effect of 3HQ derivatives on the time-course for the limited proteolysis by trypsin of human phenylalanine hydroxylase (hPAH). (**A**–**E**) Representative SDS-Polyacrylamide gel electrophoresis analyses of hPAH limited proteolysis by trypsin in the presence of 1% DMSO (**A**), or 100 µM of compounds **3** (**B**), **8** (**C**), **9** (**D**) and **11** (**E**) in 1% DMSO. (**F**) Time-course of the limited proteolysis of hPAH in the presence of 1% DMSO (●) and 100 μM of compounds **3** (■), **8** (☐), **9** (π), and **11** (○) in 1% DMSO; for each assay, the area at time zero was considered 100%; data represent the mean ± SEM (two independent experiments) and were fitted to a single exponential decay equation to determine the rate of proteolysis (min^−1^). (**G**) Effect of selected 3HQ derivatives on trypsin activity using N-CBZ-GGR-AMC as the enzyme substrate; assays were performed in the presence of 1% DMSO (●; control), and 100 μM of compound **3** (■), **8** (☐), **9** (▲), and **11** (○) in 1% DMSO; data represent the mean of three independent experiments; Trypsin activity was calculated by determining the initial velocity of the reaction using the data points presented in the graph; relative trypsin activity (considering 100% for the control) was: 79% (compound **3**; ■), 62% (compound **8**; ☐), 71% (compound **9**; ▲), and 92% (compound **11**; ○).

In order to understand whether the selected 3HQ derivatives have an impact on hPAH global conformation, we resorted to limited proteolysis by trypsin and SDS-PAGE analysis, following hPAH incubation with **3**, **8**, **9**, or **11** at 100 μM (Figure 6).

Incubation with 100 μM l-Phe in the presence of 1% DMSO decreased the proteolytic rate (*k*_P_) from 0.41 ± 0.03 to 0.15 ± 0.01 min^−1^ (Figure 6A,F) in line with our previous observation [8]. With respect to the control sample, compounds **3** and **8** exerted a protective effect as the *k*_P_ decreased to 0.29 ± 0.04 and 0.30 ± 0.01 min^−1^ (Figure 6B,C,F), respectively, whereas compounds **9** and **11** did not significantly affect the proteolytic rate (*k*_P_ of 0.38 ± 0.04 and 0.41 ± 0.01 min^−1^, respectively; Figure 6D–F). The fluorimetric control assays where a specific substrate for trypsin was used showed that none of the tested compounds inhibited trypsin activity as 79 (compound **3**), 62 (compound **8**), 71 (compound **9**) and 92% ((compound **11**) of residual trypsin activity was observed when compared to the control (Figure 6g; Supplementary Table S3).

### 3.6. Time-Dependent Thermal Stability of hPAH Activity in the Presence of Selected 3HQ Derivatives

Given the goal of discovering and developing compounds that stabilize hPAH activity, the protein was exposed to thermal stress (42 °C for different incubation periods) and the residual activity was determined at 37 °C after an equilibration period of 3 min at the same temperature [19]. As shown in Figure 4B (and Supplementary Figure S6), in the presence of compound **9**, after 60 min at 42 °C, the hPAH residual activity was 136 ± 7% of the control sample (hPAH in 1% DMSO) activity under identical conditions, and remained above 100% up to 120 min (121 ± 32% and 176 ± 32% at 90 and 120 min, respectively). Compound **11** presented a similar effect after 90-min incubation (117 ± 33%) which also increased after 120-min incubation (168 ± 64%). This protective effect was not observed for compounds **3** and **8** which, on the contrary, seem to contribute to a decrease in activity over time, and, therefore, were herein excluded from further analysis.

### 3.7. Molecular Docking of Compounds 9 and 11 to hPAH

The modulation of hPAH activity in in vitro assays by compounds **9** and **11** (Figure 4B) prompted us to perform docking simulations. Thus, these compounds were docked into hPAH active site to gain a deeper insight of the relevant interaction partners to recognition and activity. The affinity of the compounds for hPAH as well as their potential interaction with the iron center was investigated. Two crystal structures of hPAH were selected and retrieved from Protein Data Bank (3PAH and 1MMT) for this purpose. In 3PAH crystal structure, the protein is complexed with adrenaline, a catechol that is a reversible hPAH inhibitor, binding to the catalytic Fe^3+^ center through the two catechol hydroxyl moieties [36].

**Figure 7:**
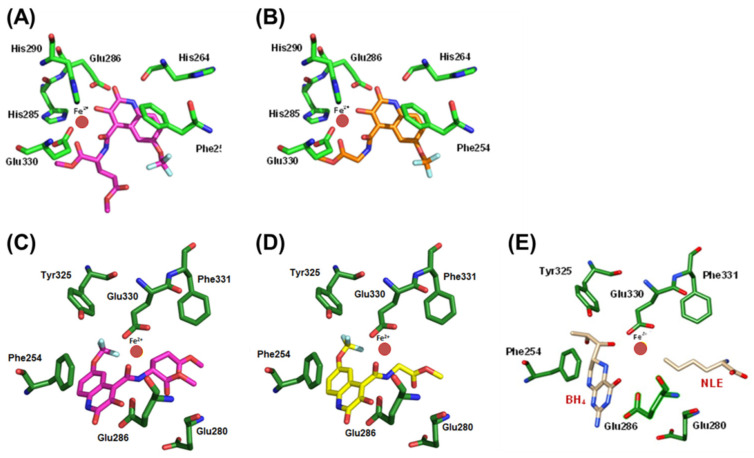
Best docking poses of selected 3HQs into human phenylalanine hydroxylase active site. (**A**,**B**) Structures of compounds **9** (**A**) and **11** (**B**) in hPAH catalytic center of PDB ID:3PAH. (**C**–**E**) Structures of compounds **9** (**C**) **11** (**D**) and BH_4_ and the substrate analogue norleucine (NLE) (**E**) in hPAH catalytic center of PDB ID:1MMT. The iron atom is depicted in brown and the most relevant interacting residues responsible for iron coordination (His285, His290 and Glu330) or establishing interactions in the binding site are pictured in the figure. Compounds show average scores of 72.98 (**9**) and 69.72 (**11**) in 3PAH and 72.87 (**9**) and 64.63 (**11**) in 1MMT.

As observed in Figure 7A,B, the 3HQs adopt similar poses inside the hPAH active site, positioning the hydroxyl group of the quinoline ring of compound **9** oriented towards the catalytic iron (at a distance of 2.15 Å; Supplementary Table S4). The fused six membered core poses of compounds **9** and **11** overlapped inside the binding pocket. For compound **11**, only the side chain in the heterocycle, 4-position, was found at distances of 1.73 and 2.25 Å from the iron ion (Supplementary Table S4). Tyr325 and Glu330 have been considered determinants for the recognition and strong binding of catecholamine inhibitors [36]. From our calculations, we observe that, in the case of compound **9**, the hydroxyl group was found at a distance of 2.66 Å to Oε2 of the Glu330 side chain (Supplementary Table S4).

To evaluate the position and orientation of the compounds in relation to the cofactor and the substrate in the catalytic pocket, we used the crystal structure of the ternary complex of hPAH with BH_4_ and norleucine (NLE), a substrate analogue (PDB ID: 1MMT). The most favored locations of the quinoline ring and the side chain of compounds **9** and **11** (Figure 7C,D) overlap significantly with the space occupied by, respectively, BH_4_ and the substrate analogue (Figure 7E). In the hPAH catalytic center, BH_4_ mainly interacts with Phe254, Leu249, Ser251, and Gly247. The most favorable poses of the selected 3HQs suggest the establishment of an aromatic π-stacking interaction with Phe254 and an H-bond with Leu249 (backbone amine), but not with Ser251 and Gly247 (backbone CO) (Supplementary Table S4). Regarding the key residues for l-Phe recognition and positioning (His285, Ser349, Arg270 and Thr278), only His285 is localized at distances of ≈2.5 Å (Supplementary Table S5), indicating availability of the above residues to recognize and bind the hPAH natural substrate. Similarly, the distances calculated between the 3HQ derivatives and the amino acid residues involved in π-stacking interactions (Pro281, Trp326, and Phe331) and the preservation of the H-bonding network with water molecules (Tyr277, Gly346, Ser350, and Glu353) necessary for substrate binding, do not point to the establishment of direct interactions. These findings suggest that the binding affinity for the 3HQs should be much lower than for the natural substrate. For both structures, the estimated binding energies (scores) calculated with the GoldScore scoring function indicate that compound **11** presented the lowest affinities (scores of 69.72 and 64.63).

### 3.8. Effect of 3HQ Derivatives on hPAH in Human Cells

**Figure 8:**
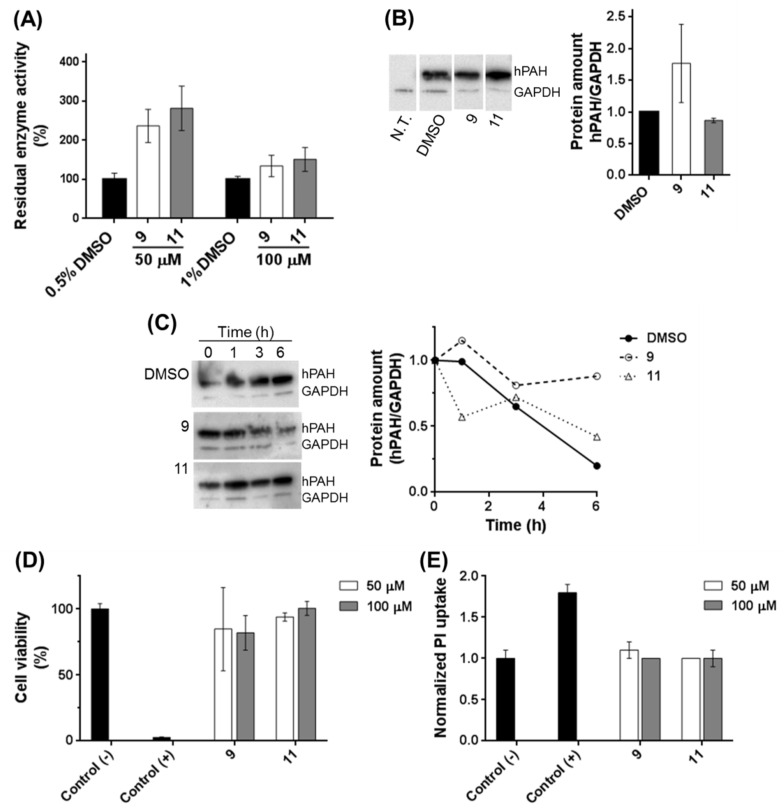
Compounds **9** and **11** increased the level and activity of human phenylalanine hydroxylase (hPAH) transiently expressed in HEK-293T cells and were shown to be biocompatible. (**A**) Effect of compounds **9** and **11** (50 and 100 μM) on hPAH activity after 24 h incubation and using DMSO (0.5 and 1%) as the control; enzyme activity was calculated as the amount of tyrosine (nmol) produced per min, per mg total protein in cell lysate and represent the mean ± SD from three independent assays each performed in triplicate. (**B**) Amount of hPAH after 24 h incubation and using 50 μM of compounds; protein amount was normalized using GAPDH as an internal control; (N.T.) Cells not transfected with expression vector. (**C**) Cells were incubated with 50 μM of compounds **9** or **11** for 24 h and protein synthesis was arrested using 10 μg/mL puromycin; cellular lysates were analyzed for protein amounts 1, 3, and 6 h after translation blockage; assays were performed in duplicate. (**D**,**E**). Cytotoxicity of 3HQ derivatives; compounds were tested at 50 and 100 μM, in HEK-293T cells, for cell viability (**D**) and propidium iodide (PI) uptake (**E**), using 1% DMSO as the negative control and sodium dodecyl sulphate (SDS) as the positive cytotoxic control; cell viability was determined by the percentage of Alamar Blue reduction (**D**); the value obtained for the negative control was considered 100%; retention rate of PI was determined relatively to the negative control which was considered 1. Data represent the mean ± SD from three independent assays each performed in triplicate. Uncropped western blot images are shown in Supplementary Figure S7.

As compounds **9** and **11** did not show competitive inhibition characteristics and presented a protective effect on hPAH activity, we performed additional studies to analyze their effect on hPAH levels and activity in cells transiently overexpressing the protein. To this end, HEK-293T cells transfected with the vector containing the hPAH-encoding cDNA were grown for 24 h in the presence of compounds **9** or **11** at 50 μM and 100 μM, using as a control 0.5 and 1% DMSO, respectively. Non-transfected cells (N.T.) were used as a negative control and did not exhibit any hPAH activity or protein expression as judged by western blots. A higher activity was observed for both compounds (Figure 8A) particularly when using 50 μM. At this concentration, whereas compound **9** in the growth medium promoted a 1.8-fold increase in hPAH expression (Figure 8B), compound **11** did not affect the hPAH protein levels. Therefore, the higher activity observed with compound **9** may be a result of a higher hPAH protein level.

Since compounds **9** and **11** (at 50 μM) seemed to protect enzyme activity, we further monitored hPAH stability as a function of time after blocking protein translation with puromycin. As observed in Figure 8C, in the presence of 50 μM of compound **9** or **11**, hPAH protein content is maintained as a function of time, as compared with control data obtained with vehicle (0.5% DMSO). This observation indicates that these compounds, particularly compound **9**, stabilize and protect hPAH from intracellular proteolysis.

The biocompatibility of the tested 3HQ derivatives was also evaluated by performing two different assays with HEK-293T cells in order to assess cell viability through metabolic activity (Alamar blue) and membrane integrity (PI). Upon incubation with the tested compounds (100 μM), the cell viability was 82 ± 13% (compound **9**) and 101 ± 5% (compound **11**) (Figure 8D). The use of 50 μM of compounds did not affect cell viability (compound **9**: 85 ± 32%; compound **11**: 94 ± 3%) (Figure 8D). At 100 μM, these compounds did not present any effect on membrane integrity (Figure 8E) as the obtained values for the relative PI uptake (compound **9**: 1.0 ± 0.0; compound **11**: 1.0 ± 0.1) were similar to the negative control (1.0 ± 0.1).

### 3.9. Effect of Compounds ***9*** and ***11*** on the Aggregation Behavior of the hPAH-RD^1–120^

**Figure 9:**
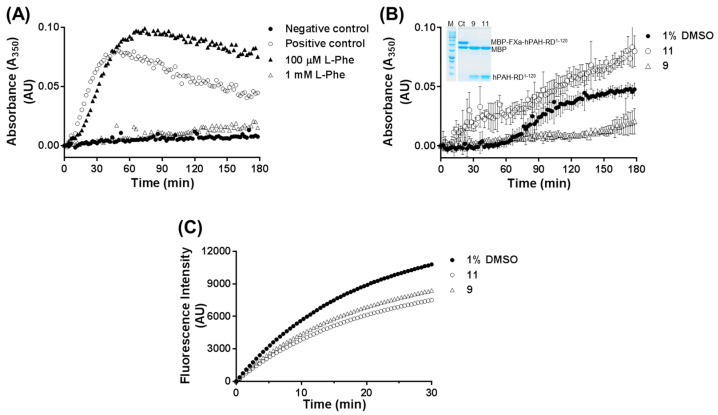
Self-association of dimeric hPAH-RD^1–120^ following cleavage of the MBP fusion protein by Factor Xa (FXa) in the absence and presence of compounds **9** and **11**. (**A**) Time-course for the FXa cleavage reaction in the absence of compounds (○; positive control) and in the presence of 100 μM (▲) and 1 mM l-Phe (△); (●) dimeric MBP-FXa-hPAH-RD^1–120^ fusion protein in the absence of FXa (negative control); errors bars were omitted for clarity. (**B**) Time-course for the reaction in the presence of 1% DMSO (●) and 100 μM of compounds **9** (△) and **11** (○); the inset panel represents the SDS-PAGE analysis of a control sample (Ct; consisting of MBP-FXa-hPAH-RD^1–120^ (≈53 kDa) and MBP (≈40 kDa)) and cleaved hPAH-RD^1–120^ (≈13 kDa) at time 180 min in the presence of compounds **9** and **11**. (**C**) Effect of selected 3HQ derivatives on FXa activity using Boc–Ile–Glu-Gly–Arg–AMC as the enzyme substrate; assays were performed in the presence of 1% DMSO (●; control), and 100 μM of compound **9** (△) and **11** (○) in 1% DMSO; data represent the mean of three independent experiments; FXa activity was calculated by determining the initial velocity of the reaction using the data points presented; FXa relative enzyme activity (considering 100% for the control) was 77% (compound 9; △) and 69% (compound 11; ○).

In order to understand the observed effects on the kinetic parameters and intracellular stability of hPAH allostery in the presence of compounds **9** and **11**, we used an hPAH truncated version consisting solely of the N-terminal regulatory domain (amino acids 1–120), which has been posited to harbor the allosteric l-Phe binding site [4,6]. Although dimeric hPAH-RD^1–120^ is very unstable and presents a high tendency to aggregate, it is stabilized in the presence of 1 mM l-Phe (Figure 9) [17].

This tendency to aggregate is circumvented by producing recombinant hPAH-RD^1–120^ fused to MBP. In this form, hPAH-RD^1–120^ is stable (Figure 9A; negative control) whereas, upon cleavage of the MBP tag by FXa, the protein aggregates (Figure 9A; positive control).

As shown in Figure 9A, whereas 100 µM l-Phe only slightly prevented hPAH-RD^1–120^ aggregation, 1 mM l-Phe completely blocked it. Notably, compound **9** (100 µM) exerted a similar preventive hPAH-RD^1–120^ aggregation (Figure 9B). The absence of aggregation due to inhibition of FXa activity was ruled out by monitoring the protein species at the end of the reaction by SDS-PAGE analysis (inset to Figure 9B) and by enzymatic assays using an FXa synthetic substrate (Figure 9C). Using this enzymatic reaction, 77 and 69% FXa residual enzyme activity was retained in the presence of compounds **9** and **11**, respectively (Supplementary Table S3).

### 3.10. Effect of Compound ***9*** on the Conformational Changes of hPAH in Solution

**Figure 10:**
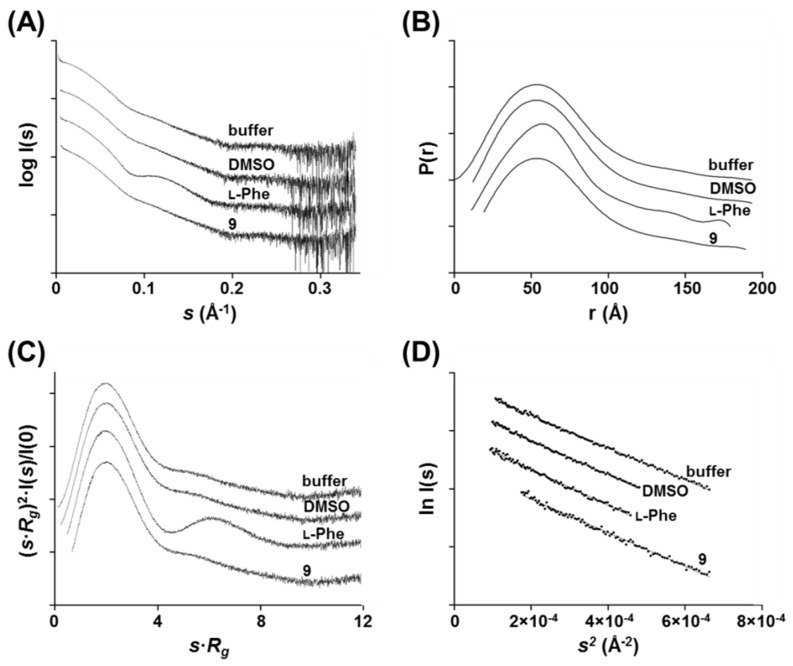
Effect of compound **9** on the small-angle X-ray scattering (SAXS) profile of human phenylalanine hydroxylase (PAH). SAXS data was collected in static mode in 20 mM Na-HEPES, 200 mM NaCl, pH 7.0 (Buffer), in buffer/2% DMSO (DMSO), in buffer/2% DMSO/200 μM l-Phe (l-Phe) and in buffer/2% DMSO/200 μM compound **9** (9). (**A**) SAXS curves of hPAH; the minimum at s~0.1 Å^−1^ in the l-Phe sample is consistent with domain rearrangement, not observable in both the ‘resting state’ (Buffer and DMSO conditions) nor in the presence of compound **9**. (**B**) Pair-distribution functions P*(r)* derived from the scattering profiles and representing the sample in the real-pace; hPAH in the presence of compound **9** displays an identical profile to the ‘resting state’, distinct from the ‘activated state’ (l-Phe condition) with decreased *D*_max_. (**C**) Kratky plots of hPAH, indicative of the enzyme’s flexibility degree; hPAH in the presence of compound **9** behaves as the ‘resting state’, unlike l-Phe-activated hPAH whose sharper decay to zero is consistent with a more rigid and less flexible particle. (**D**) Guinier representation of hPAH scattering profiles showing linearity within the Guinier region.

Given the effect of compound **9** on the aggregation behavior of hPAH regulatory domains, we used SAXS to search for ligand-induced global conformational changes of these domains, similar to those observed upon binding of the allosteric activator l-Phe. Data were collected for hPAH in the absence and presence of 200 μM l-Phe or 200 μM of compound **9**. The scattering profiles and extracted parameters are displayed in Figure 10 and Table 2, respectively.

The presence of DMSO did not influence the solution scattering behavior of hPAH (buffer vs. DMSO conditions). The higher than expected [8] structural parameters (namely *R*_g_ and *D*_max_, and the excluded particle volume, *V*_P_) (Table 2) suggest the presence of higher oligomeric states and/or aggregates in solution, particularly for the l-Phe condition, despite careful manipulation of the isolated tetrameric form. This observation probably reflects the fact that the tendency of hPAH to equilibrate between different oligomeric states disfavors the batch mode of SAXS data collection in comparison with SEC-SAXS [8,37]. However, the computed molecular masses and the linearity of the Guinier plots for *sR* < 1.3 (Figure 10D) indicate that the tetramer is the major oligomeric species rather than larger aggregates. Despite the higher contribution of aggregates in the l-Phe condition, the effect of allosteric activation on hPAH global conformation is obvious: the adoption of a more compact structure upon dimerization of regulatory domains is evidenced by (i) the appearance of a minimum at *s* ~ 0.1 Å^−1^ in the scattering profile (Figure 10A), (ii) the disappearance of extended elements in the pair distribution function with a decrease in *D*_max_ (Figure 10B), and (iii) the sharper decay to zero in the Kratky plot (Figure 10C). Thus, a comparative analysis of the scattering profile, real-space representation, flexibility degree and extracted parameters of hPAH in the different conditions can indicate the adoption of either a ‘resting’ or an ‘active’ state. The identical behavior of the DMSO and compound **9** samples suggests that this 3HQ derivative does not induce the adoption of an ‘active’ conformation with dimerized regulatory domains, thus binding at a different location than the allosteric l-Phe binding site. Indeed, a fitting analysis of the compound **9** SAXS experimental curve with SAXS models of hPAH in the ‘resting’ (SASDFB7) and ‘active’ (SASDFC7) conformations confirms the good agreement with the ‘resting state’ (Supplementary Figure S8).

## 4. Discussion

Due to the high number of different pathogenic mutations identified in the PAH gene (>1000), development of a ‘universal’ molecule aiming at correcting the activity and/or stability of the entire spectrum of protein variants appears to be unrealistic. Therefore, the discovery of different classes of small molecules modulators of hPAH folding and/or activity will contribute to the development of pharmacological strategies to treat a larger cohort of PKU patients. Random searches of large compound libraries using high-throughput screenings (HTS) or shape-focused virtual screenings have been used to identify small molecules targeting hPAH [34,38]. Herein, a different approach was applied based on compounds inspired by functional and/or structural characteristics of the enzymes’s catalytic site, namely its substrate l-Phe, the BH_4_ cofactor and the catalytic non-heme ferric center. Using this rationale, we evaluated a library of 20 compounds with 3HQ as the core structure displaying the ability to complex iron, a structural similarity to BH_4_ and, for some molecules, l-Phe-like motifs as substituent groups were present. Our goal was to identify 3HQ derivatives able to potentiate/stabilize enzyme structure and activity without significantly inhibiting the enzyme by outcompeting the natural substrate, and still allowing further activation by l-Phe in response to a potentially neurotoxic circulating substrate overload.

Among the 20 3HQ derivatives, **3**, **8**, **9**, and **11** were selected as the most promising candidate molecules. None of these compounds presented inhibitory effects on hPAH activity in the non-activated condition, while compound **3** even afforded a mild increase. Importantly, they all allowed further activation by l-Phe with respect to the pre-activated condition control. This is an especially relevant characteristic since often active variants with close-to-WT activity exhibit disturbed allosteric regulation as the underlying pathogenic mechanism [21], both in PKU and other IMD (e.g., in [39,40]). The changes observed in the EPR spectra provided evidence that these four compounds establish interactions with the catalytic iron. However, the modest decrease in enzymatic activity does not foresee a tight binding of the 3HQs to the iron center as that observed for catecholamines such as dopamine, noradrenaline, and adrenaline [36]. These molecules are potent inhibitors of hPAH activity with a high affinity binding (*K*_D_ < 1 μM) and their effect has been associated with a tight direct coordination of Fe^3+^ [36]. As for the selected 3HQs derivatives, the EPR studies suggest different modes of iron interaction, as judged from the shape and intensity of the main signal centered at g = 4.25 and from the additional resonances that appeared as a consequence of compound incubation. Compound **3** promoted an increase in the g = 4.25 signal intensity, in contrast to the decrease promoted by catecholamines [39]. While for all compounds the resonances observed at g ≈ 9.2 and at g ≈ 5.1–5.3 are possibly related with some degree of flexibility of the iron coordination geometry (which includes three waters) [35], a less rhombic symmetry was observed for compounds **8**, **9**, and **11**, suggesting the exclusion of an iron-bound water molecule from iron coordination. Similar observations have been previously reported for hPAH incubated with its cofactor BH_4_, dopamine [35], noradrenaline (in the absence or presence of oxygen), or prepared in the hydroxyl moiety rich Tris buffer (shown to compete with the cofactor analogue 6-methyl-5,6,7,8-tetrahydrobiopterin for the active site) [41]. Structural studies have shown that dopamine and other catecholamines eliminate two of the three iron-coordinating waters, in line with the increase in less rhombic signals at higher g values observed by EPR [35,36,41]. Herein, the EPR studies suggest that the 3HQ derivatives may exert a similar effect of replacing water ligands from the iron center through the hydroxyl moiety from the hydroxyquinoline ring system. The comparative analysis of the estimated *K*_D_ (in the absence of l-Phe) obtained by SPR suggests higher affinities for compounds **3** and **8** than for **9** and **11**. In addition, steady-state kinetics studies characterized the effect of compounds **3** and **8** as competitive inhibitors towards the l-Phe substrate. We posit that the phenylalanine moiety present in the structures of compounds **3** and **8** contributes to the higher affinity for the protein and direct competition with the substrate l-Phe, as compared to those of compounds **9** and **11**, which contain instead the carbomethoxy derivatives of L-glutamate and L-glycine, respectively.

Evaluation of the time-dependence of full-length hPAH degradation by trypsin has been a valuable tool to study changes in hPAH global conformation occurring upon pre-incubation with the substrate or in the presence of the BH_4_ cofactor [4,8,42]. Allosteric l-Phe binding protects rat PAH (rPAH) from trypsin digestion [43] as the rotational movement of the N-terminal domain, necessary for their dimerization, and l-Phe allosteric binding decreases the accessibility of the C-terminal part of this domain that is highly prone to trypsin digestion. A similar protective effect has been reported for BH_4_ binding to the hPAH catalytic center [42] by a mechanism involving H-bonding between the two hydroxyl groups of BH_4_ and Ser23 located in the regulatory domain and formation of a more closed conformation, which is less prone to trypsin digestion [4]. The protective effect of compounds **3** and **8** against hPAH proteolysis may be caused by a tighter binding to the catalytic pocket, thus promoting the stabilization of a more closed structure rather than inducing dimerization of the N-terminal domain, as these compounds did not show any ability to pre-activate the enzyme.

The absence of *k*_P_ proteolytic rates variations in the presence of compounds **9** or **11** argues against strong global conformational changes of hPAH. The steady-state kinetic parameters (absence of competitive inhibition) obtained for these compounds suggests that, besides binding to the catalytic domain (EPR assays), compounds **9** and **11** will also bind to another region of the protein. Of note is the fact that only compounds **9** and **11** were able per se to pre-activate the enzyme (Figure 3A; NA vs. 3HQ-A), indicating a higher exposure of the catalytic pocket in the presence of these compounds. Corroborating this hypothesis, the data obtained for compound **9** on the aggregation behavior of the N-terminal regulatory domain (hPAH-RD^1–120^) strongly suggests that this molecule is able to bind to this domain, probably in a region different from the l-Phe allosteric site. Indeed, in the presence of compound **9**, hPAH allostery was maintained (Table 1) and the protein was able to be further activated by l-Phe (Figure 3A). Moreover, solution scattering data of hPAH in the presence of compound **9** reveals no gross structural changes consistent with domain dimerization as in the l-Phe allosteric activation mechanism [8,37], suggesting a global conformation similar to the enzyme’s ‘resting state’. Concerning compound **11**, no direct evidence of a direct binding to hPAH-RD^1–120^ was obtained, although binding to other regions of the protein besides the active site could not be excluded. Indeed, among the selected 3HQ derivatives, only compounds **3** and **11** afforded a slight stabilization of the regulatory domain. This may result from the secondary binding to another site within the protein, which may propagate and indirectly affect the regulatory domain stability. Upon exposure of isolated hPAH to a thermal insult, compounds **9** and **11** were those that most contributed to retaining the enzymatic activity at long incubation times. Further studies on HEK-293T cells showed that, also in a cellular environment, compounds **9** and **11** were able to stabilize the protein leading to a higher hPAH content and activity. The preservation of enzyme activity also suggests a proper assembly of the biologic tetrameric forms. Taken together, these data indicate that an appropriate screening strategy to identify hPAH stabilizers should rely both on hPAH activity protection and conformational and thermal stability.

From our series of 3HQ derivatives, compounds **9** and **11** could be regarded as promising hit molecules for the development of a new class of molecules acting as activity chaperones protecting hPAH activity. The majority of the experimental approaches aiming at the identification of pharmacological chaperones target the enzyme catalytic center. From this perspective, enzyme inhibitors are often included in the group of molecules that upon binding to the active site promote protein stabilization, thus acting as pharmacological chaperones [44,45], alike sapropterin dihydrochloride being employed for hPAH clinical variants. Upon hPAH binding, BH_4_ establishes interactions with residues of the N-terminal regulatory domain, promoting a less flexible and more closed structure with a lower enzymatic activity but less prone to unfolding and degradation [11]. In addition, enzyme inhibitors have also been described as protectors of enzyme activity of human tyrosine hydroxylase [46,47], a protein that shares with hPAH several structural and catalytic properties. Binding of l-Phe to the allosteric site stabilizes an ‘activated’ state necessary for the physiological response to toxic levels of l-Phe. Therefore, the ideal stabilizing compound should bind to a protein region other than the active site and the allosteric site [7]. In this respect, compound **9** is a starting structure for the discovery of such a molecule. It is well-established that compounds envisaged to be used as stabilizers of protein structure/activity should bind specifically to the target molecule but should also easily dissociate from the enzyme in the presence of the substrate, allowing the protein to exert its biological function [3]. In line with this observation, the selected 3HQs presented lower affinities than those obtained for the substrate l-Phe. Finally, an important feature of a molecule intended to be used as a pharmacological therapy is their effect in a cellular context and their biocompatibility. Compounds **9** and **11** were devoid of toxicity and, importantly, preserved the hPAH content and concomitantly enzyme activity. These compounds, besides representing strong candidates for structure optimization aiming at improved properties, also provided proof-of-concept for the utilized strategy of compound design directed to a catalytic center that can be applied to other deficient enzymes responsible for IMDs.

## Figures and Tables

**Table 1:** Steady-state kinetic parameters of human phenylalanine hydroxylase (hPAH) for the substrate (l-Phe) in the absence and presence of 100 μM of selected 3HQ.

	*V*_max_ (10^3^ nmol Tyr.min^−1^.mg^−1^)	*S*_0.5_ (µM)	*k_i_* (mM)	*h*	*k*_cat_/*S*_0.5_ (µM^−1^/min)	Activation Ratio ^4^
Buffer ^1^	4.89 ± 0.34	98 ± 15	22.2 ± 3.4	1.8 ± 0.2	2.7	1.8
1% DMSO ^1^	4.17 ± 0.14	81 ± 4	8.8 ± 1.4	2.1 ± 0.2	2.8	1.7
Compound **3** ^3^	4.82 ± 0.64	206 ± 99 *	-	-	1.3	1.3
Compound **8** ^1^	4.38 ± 0.94	115 ± 9 *	7.2 ± 0.2	1.2 ± 0.3	2.1	1.7
Compound **9** ^1^	3.94 ± 0.64	108 ± 34	23.3 ± 0.5	1.9 ± 0.8	2.0	1.4
Compound **11** ^2^	7.75 ± 1.23 **	386 ± 97 **	2.5 ± 0.3	-	2.7	1.5

Enzyme activity measured at 25 °C in the presence of 75 μM BH_4_ and variable concentrations of l-Phe (25 μM to 2.5 mM). (*S*_0.5_) l-Phe concentration at half-maximal activity; (*h*) Hill coefficient; (*k*_cat_/*S*_0.5_) catalytic efficiency calculated considering the MM of a hPAH subunit (55 kDa). *p* (* < 0.01; ** < 0.0001) calculated comparing compound values with 1% DMSO. Data fitted to the modified equations of ^1^ Hill [18] (Equation (1)), ^2^ Michaelis–Menten (Equation (2)) accounting for substrate inhibition and the ^3^ Michaelis–Menten equation (no substrate inhibition). ^4^ Ratio of l-Phe activation was obtained using 100 μM substrate. Values represent the mean ± SD of three independent assays.

**Table 2:** Small-angle X-ray scattering (SAXS) structural parameters of human phenylalanine hydroxylase (hPAH).

	*R*_g_^Guinier^ (Å)	*R*_g_^P(r)^ (Å)	*D*_max_ (Å)	*V*_P_ (Å^3^)	MM (kDa)	MM_expected_ (kDa)
Buffer	49.43 ± 0.04	51.46 ± 0.06	193	423,841	265	223
DMSO	50.31 ± 0.71	52.16 ± 0.06	193	427,960	267	223
l-Phe	52.73 ± 0.07	52.25 ± 0.04	179	629,301	393	223
Compound **9**	52.14 ± 0.07	52.00 ± 0.05	189	428,899	268	223

SAXS data was collected in static mode in buffer (20 mM Na-HEPES, 200 mM NaCl, pH 7.0), in buffer/2% DMSO, in buffer/2% DMSO/200 μM l-Phe and in buffer/2% DMSO/200 μM compound **9**. Radii of gyration (*R*_g_) were estimated from the Guinier approximation and the pair-distribution function *P(r)*. Maximum particle dimensions (*D*_max_) were obtained from the pair-distribution function. Excluded particle volumes (*V*_P_) were estimated from the Porod approximation. Molecular mass (MM) values were estimated as MM = *V*_P_/1.6. The expected molecular mass (MM_expected_) was estimated based on the protein primary sequence. For more details, see Materials and Methods and Supplementary Table S6.

## Data Availability

All data are available upon reasonable request to the corresponding authors.

## Supplementary Materials

The following are available online at https://www.mdpi.com/2218-273X/11/3/462/s1, Figure S1: Size exclusion chromatography (SEC) of recombinant full-length human phenylalanine hydroxylase, obtained from IMAC purification, Figure S2: Size exclusion chromatography (SEC) of the MBP-FXa-hPAH-RD^1–120^ protein obtained from amylose purification, Figure S3: Schematic representation of the three conditions tested for the enzymatic assays of human phenylalanine hydroxylase, Figure S4: Representative thermal unfolding profiles of human phenylalanine hydroxylase (hPAH) monitored by differential scanning fluorimetry, Figure S5: Electron paramagnetic resonance (EPR) spectra of control samples, Figure S6: Time-dependent thermal inactivation profile of human phenylalanine hydroxylase in the absence and presence of selected 3HQ derivatives, Figure S7. Expression profiles of human phenylalanine hydroxylase in lysates of HEK293T cells analyzed by immunoblotting and presented in manuscript Figure 7B,C, Figure S8: Superposition of human phenylalanine hydroxylase (hPAH) SAXS experimental curves with SASBDB models of ‘resting’ and ‘active’ states of hPAH, Table S1: *T*_m_ values calculated by differential scanning fluorimetry, Table S2: Electron paramagnetic resonance (EPR) parameters of control samples, Table S3: Trypsin and Factor Xa (FXa) activity in the presence of 1% DMSO and 100 µM of selected compounds, Table S4: Calculated distances (Å) of the selected 3HQs to the catalytic non-heme iron and its ligands (His285, His290 and Glu330), and to specific amino acid residues that interact with catecholamines reversible inhibitors (Phe254, Tyr325 and Glu330), Table S5: Calculated distances (Å) of the selected 3HQs to specific amino acid residues, involved in cofactor (BH_4_) and substrate (l-Phe) binding of human phenylalanine hydroxylase, Table S6: Small-angle X-ray scattering data collection and experimental parameters for human phenylalanine hydroxylase (hPAH) with references [48,49,50]; Annex: Compound synthesis and analysis.
