# Supplementary material for: Modulation of Human Phenylalanine Hydroxylase by 3-Hydroxyquinolin-2(1H)-One Derivatives
Source: Biomolecules. 2021 Mar 19;11(3):462. doi: 10.3390/biom11030462 (PMC8003416; doi:10.3390/biom11030462)
Supplement: Supplementary file 1 [file biomolecules-11-00462-s001.pdf]

SUPPLEMENTARY MATERIALS

Modulation of human phenylalanine hydroxylase by 3-hydroxyquinolin-2(1H)-one derivatives

Raquel R. Lopes, Catarina S. Tomé, Roberto Russo, Roberta Paterna, João Leandro, Catarina S. Tomé, Nuno R. Candeias, Lídia M. D. Gonçalves, Miguel Teixeira, Pedro M. F. Sousa, Rita C. Guedes, João B. Vicente, Pedro M. P. Gois, Paula Leandro

TABLE OF CONTENTS

Figure S1. Size exclusion chromatography (SEC) of recombinant full-length human phenylalanine hydroxylase, obtained from IMAC purification.
Figure S2. Size exclusion chromatography (SEC) of the MBP-FXa-hPAH-RD$^{1–120}$ protein obtained from amylose purification.
Figure S3. Schematic representation of the three conditions tested for the enzymatic assays of human phenylalanine hydroxylase (hPAH). Non-activated (NA); Compound-activated (CA) and Compound/Substrate activated (CSA).
Figure S4. Representative thermal unfolding profiles of human phenylalanine hydroxylase (hPAH) monitored by differential scanning fluorimetry.
Figure S5. Electron paramagnetic resonance (EPR) spectra of control samples containing 100 µM $FeCl_3$ solutions incubated with equimolar amounts of the selected 3HQs derivatives.
Figure S6. Time-dependent thermal inactivation profile of human phenylalanine hydroxylase in the absence and presence of selected 3HQ derivatives.
Figure S7. Expression profiles of human phenylalanine hydroxylase (hPAH) in lysates of HEK293T cells analysed by immunoblotting and presented in manuscript Figure 8C (A) and Figure 8D (B).
Figure S8. Superposition of human phenylalanine hydroxylase (hPAH) SAXS experimental curves with SASBDB models of 'resting' and 'active' states of hPAH.
Table S1. Tm values calculated by Differential scanning fluorimetry (DSF)
Table S2. Electron paramagnetic resonance (EPR) parameters of control samples
Table S3. Trypsin and Factor Xa (FXa) activity in the presence of 1% DMSO and 100 µM of selected compounds.
Table S4. Calculated distances (Å) of the selected 3HQs to the catalytic non-heme iron and its ligands (His285, His290 and Glu330), and to specific amino acid residues that interact with catecholamines reversible inhibitors (Phe254, Tyr325 and Glu330)
Table S5. Calculated distances (Å) of the selected 3HQs to specific amino acid residues, involved in cofactor (BH4) and substrate (l-Phe) binding of human phenylalanine hydroxylase
Table S6. Small-angle X-ray scattering data collection and experimental parameters for hPAH with references [48-50].

ANNEX

Compound synthesis and analysis

1. General Remarks
2. Synthesis of *N*-hydroxysuccinimide (NHS) – Diazoacetate 15
3. Synthesis of 4-NHS-3-hydroxyquinolin-2(1H)-ones 16 and 17
4. Synthesis of Phe-3HQs 1 and 2

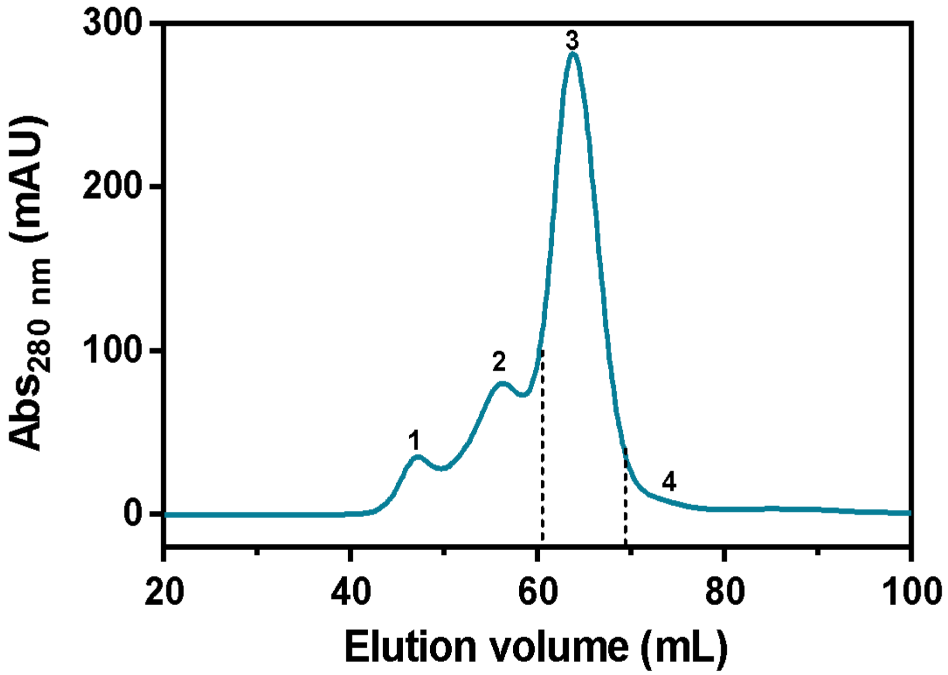

Figure S1. Size exclusion chromatography (SEC) of recombinant full-length human phenylalanine hydroxylase, obtained from IMAC purification. Peak 1 represents higher-order oligomeric forms (eluted in the void volume), peak 2 a presumably octameric form, peak 3 the tetramer (~220 kDa) and peak 4 the dimer (~110 kDa). The apparent molecular mass of the enzyme forms were estimated using the elution position of standard molecular mass markers as a reference (not shown). SEC was performed on a HiLoad Superdex 200 HR column (1.6 cm 9 60 cm; GE Healthcare), equilibrated and eluted with 20 mM Na-Hepes, 200 mM NaCl, pH 7.0 at a flow rate of 0.7 mL.min^{-1}, at 4 °C and detection at 280 nm.The two dashed lines indicate the collected tetramers utilized for the assays.

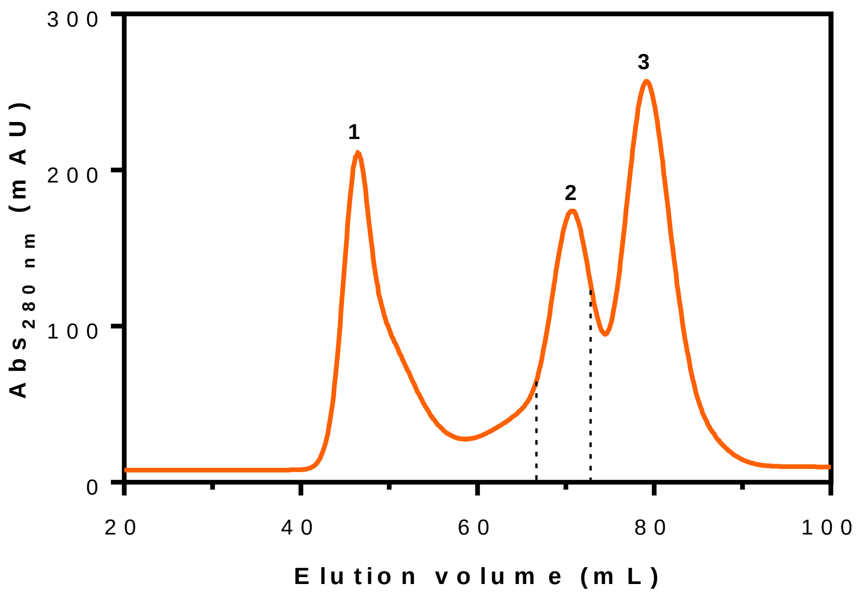

Figure S2. Size exclusion chromatography (SEC) of the MBP-FXa-hPAH-RD$^{1–120}$ protein obtained from amylose purification. Peak 1, higher order oligomeric forms (eluted at the void volume); peak 2, dimeric form (~ 156 kDa); peak 3, monomeric form (~ 65 kDa). The molecular mass of the enzyme forms were estimated using the elution position of standard molecular mass markers as a reference (not shown). SEC was performed on a HiLoad Superdex 200 HR column (1.6 cm 9 60 cm; GE Healthcare), equilibrated and eluted with 20 mM Na-Hepes, 200 mM NaCl, pH 7.0 at a flow rate of 0.7 mL.min^{-1}, at 4 °C and detection at 280 nm. The two dashed lines indicate the collected dimers utilized for the assays.

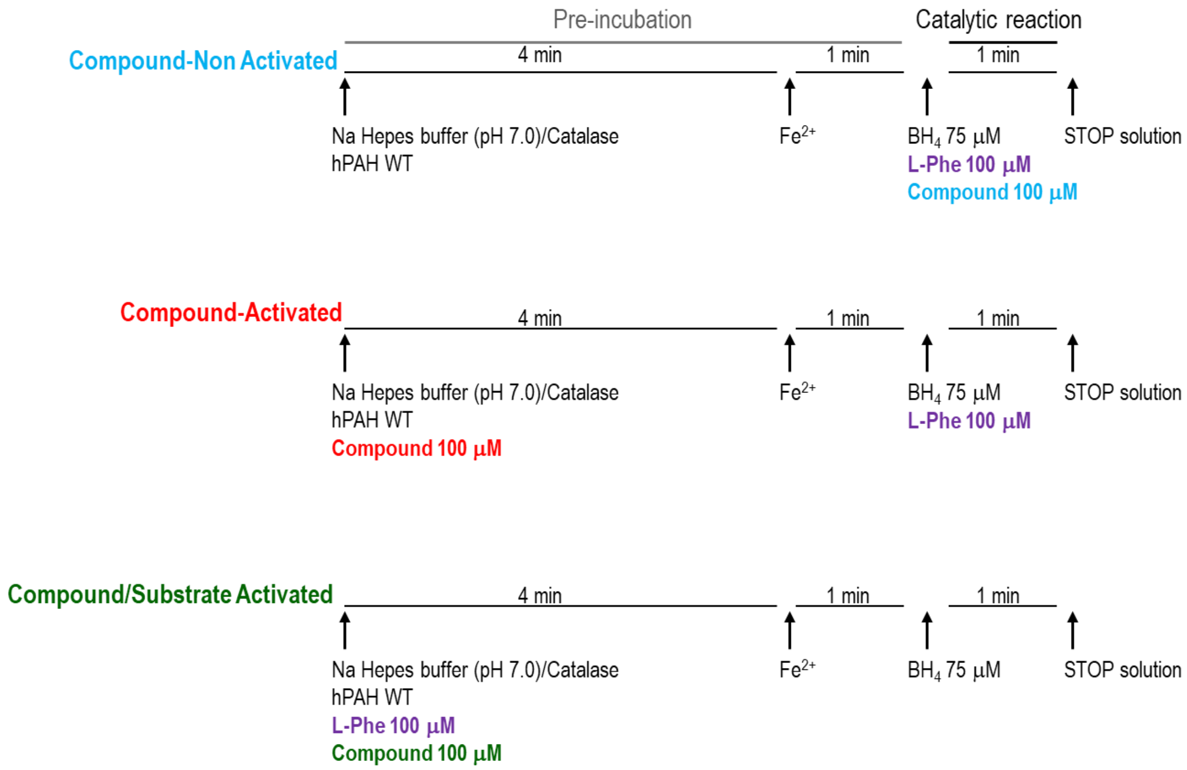

Figure S3. Schematic representation of the three conditions tested for the enzymatic assays of human phenylalanine hydroxylase (hPAH). Compound Non-activated (C-NA); Compound-activated (C-A) and Compound/Substrate Activated (CS-A). Reaction was performed at 25 ºC, except for the time-dependent inactivation assays where the reaction was performed at 37 ºC upon pre-incubation with the compounds (100 µM) or 1% DMSO, at 42 ºC.

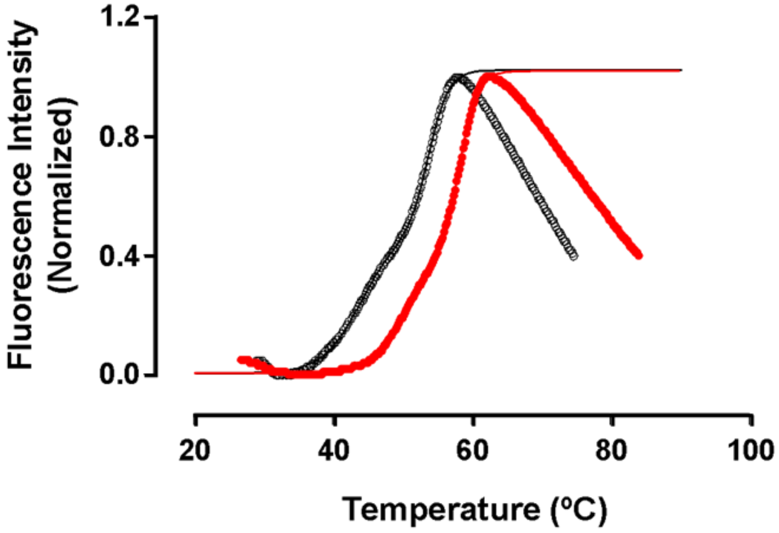

Figure S4. Representative thermal unfolding profiles of human phenylalanine hydroxylase (hPAH) monitored by differential scanning fluorimetry. Assays were performed in SEC buffer containing 1% DMSO in the absence (**O**) and presence (**O**) of 1 mM L-Phe. Data represent the mean of three independent experiments. Error bars are not shown for clarity. The hPAH tetramer was used at 100 µg·mL^{-1}.To determine the temperature of denaturation of the regulatory (T_{m1}) and catalytic (T_{m2}) domains (midpoints of the transitions) the data points were fitted to a biphasic equation using GraphPad Prism (version 6.01) (continuous lines).

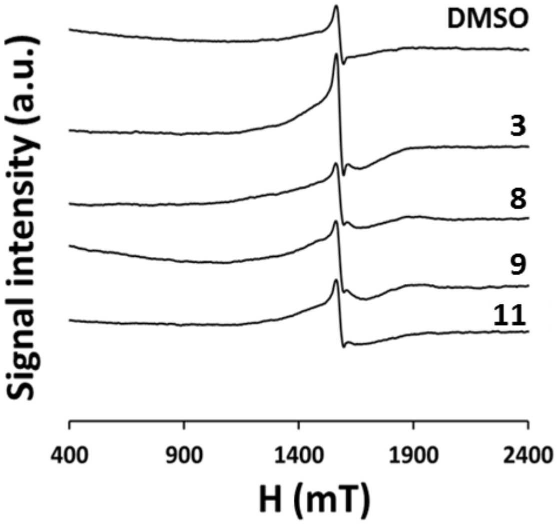

Figure S5. Electron paramagnetic resonance (EPR) spectra of control samples containing 100 µM $FeCl_3$ solutions incubated with equimolar amounts of the selected 3HQs derivatives. The spectra of compounds **3**, **8**, **9** and **11** were recorded at 4K in a Bruker EMX spectrometer equipped with an Oxford Instruments ESR-900 continuous flow helium cryostat. Microwave frequency: 9.39 GHz; microwave power, 2 mW; modulation amplitude, 1 mT.

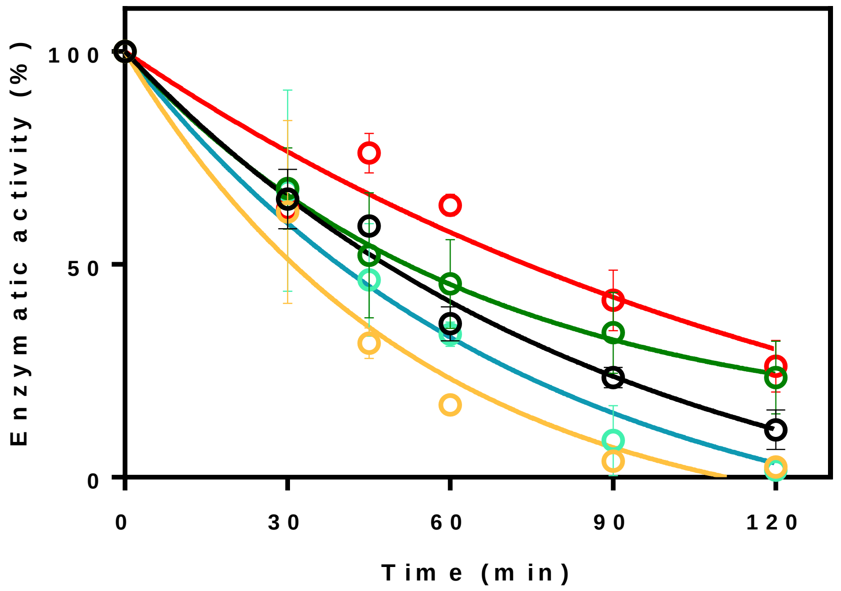

Figure S6. Time-dependent thermal inactivation profile of human phenylalanine hydroxylase in the absence and presence of selected 3HQ derivatives. Protein residual enzymatic activity was determined after 30, 45, 60, 90 and 120 min pre-incubation at 42 °C in the presence of 1% DMSO (control) and 100 μM of selected compounds. The enzymatic activity obtained at time zero for the control (—O—), compound **3** (—O—), **8** (—O—), **9** (—O—) and **11** (—O—) was considered 100%. Data represent $\overline{X} \pm SD$ from three independent assays.

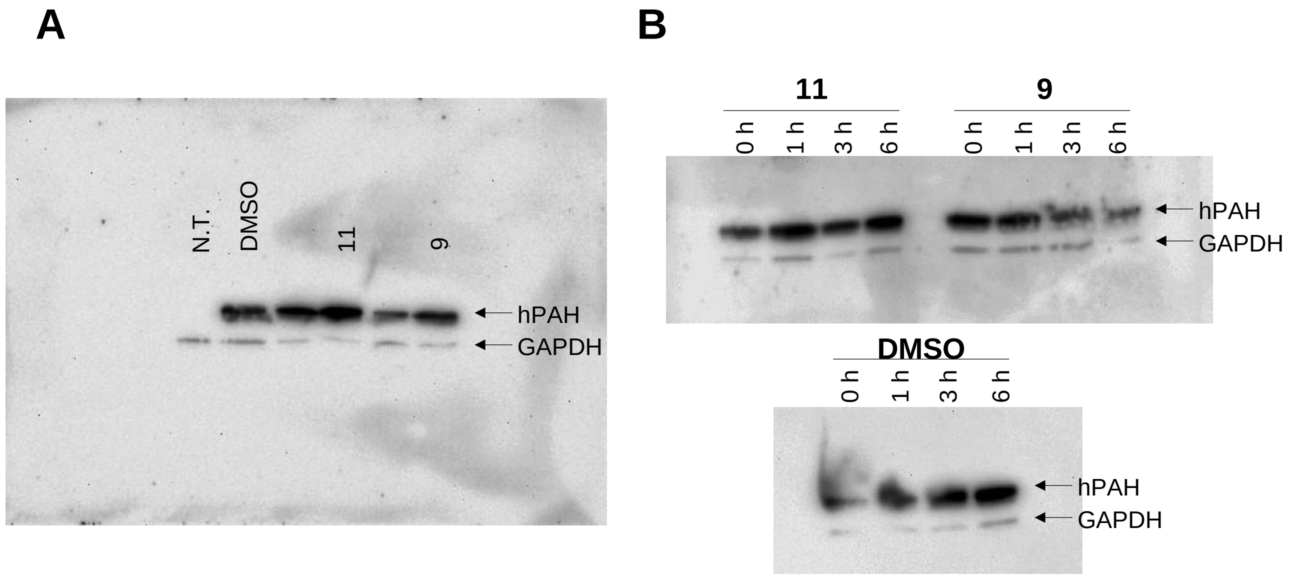

Figure S7. Expression profiles of human phenylalanine hydroxylase (hPAH) in lysates of HEK293T cells analysed by immunoblotting and presented in manuscript Figure 8B (A) and 8C (B).

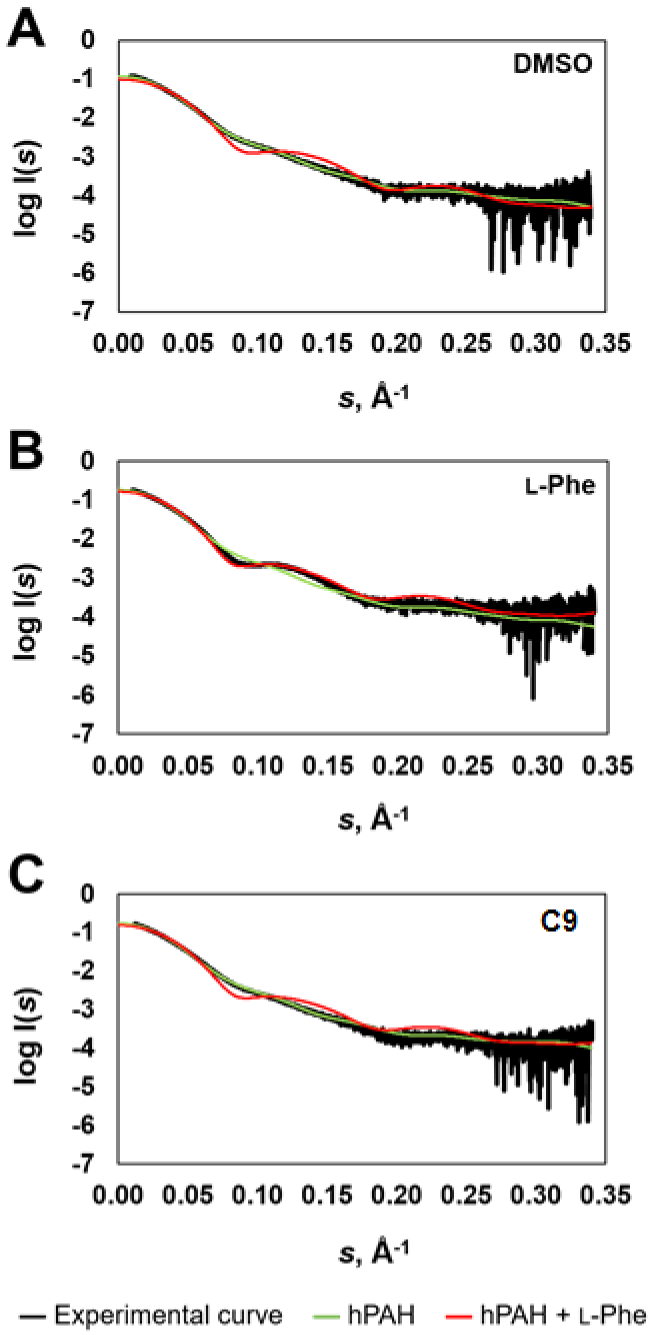

Figure S8. Superposition of human phenylalanine hydroxylase (hPAH) SAXS experimental curves with SASBDB models of 'resting' and 'active' states of hPAH. Fitting analysis was performed with CRYSOL using SASBDB entries SASDFB7 and SASDFC7, corresponding to hPAH in the absence (—) and presence (—) of 1 mM L-Phe, respectively. The experimental curves of hPAH (—) correspond to conditions (A) 2% DMSO, (B) 200 μM L-Phe in 2% DMSO and (C) 200 μM of compound **9** in 2% DMSO.

Table S1. T_m values, and corresponding ΔT_m obtained by Differential Scanning Fluorimetry (DSF) for the regulatory (R) and catalytic (C) domains of human phenylalanine hydroxylase.

	Regulatory Domain		Catalytic Domain	
**Compound**	$\boldsymbol{T_{m1}}$ **(°C)**	$\boldsymbol{\Delta T_{m1}}$ **(°C)**	$\boldsymbol{T_{m2}}$ **(°C)**	$\boldsymbol{\Delta T_{m2}}$ **(°C)**
**1% DMSO**	43.4 ± 0.6	-	53.5 ± 0.4	-
**1**	39.4 ± 1.0	-3.9 ± 0.10	55.9 ± 0.2	+2.4 ± 0.01*
**2**	35.6 ± 0.2	-7.8 ± 0.04	43.0 ± 0.4	-10.5 ± 0.11
**3**	51.7 ± 0.4	+8.3 ± 0.06	53.6 ± 0.2	+0.1 ± 0.01*
**4**	37.8 ± 1.9	-5.6 ± 0.28	42.7 ± 0.3	-10.8 ± 0.08
**5**	38.7 ± 0.2	-4.7 ± 0.02	42.3 ± 0.2	-11.2 ± 0.05
**6**	42.5 ± 0.2	-0.9 ± 0.01*	53.0 ± 0.1	-0.5 ± 0.01*
**7**	43.3 ± 0.1*	-0.1 ± 0.01*	53.7 ± 0.1*	+0.2 ± 0.01*
**8**	43.3 ± 0.1	-0.1 ± 0.01*	52.2 ± 0.3	-1.3 ± 0.01*
**9**	43.5 ± 0.1	+0.2 ± 0.01*	53.0 ± 0.1*	-0.5 ± 0.01*
**10**	43.1 ± 0.5	-0.3 ± 0.01*	53.4 ± 0.2	-0.1 ± 0.01*
**11**	47.4 ± 0.1	+4.0 ± 0.01*	53.6 ± 0.2	-0.1 ± 0.01*
**12**	43.8 ± 0.1	0.4 ± 0.01	53.1 ± 0.1*	-0.4 ± 0.01*
**13**	44.6 ± 0.4	+1.2 ± 0.01	53.5 ± 0.1	+0.1 ± 0.01*
**14**	44.8 ± 0.1	+1.4 ± 0.01*	53.5 ± 0.1	+0.1 ± 0.01*
**15**	44.5 ± 0.8	+1.1 ± 0.02	53.3 ± 0.3	-0.2 ± 0.01*
**16**	45.0 ± 1.9	+1.7 ± 0.07	53.2 ± 0.8	-0.3 ± 0.01*
**17**	44.6 ± 0.2	+1.2 ± 0.01*	53.7 ± 0.2	+0.2 ± 0.01*
**18**	40.0 ± 1.0	-3.4 ± 0.09	44.0 ± 1.4	-9.6 ± 0.31
**19**	33.0 ± 0.3	-10.4 ± 0.09	35.5 ± 0.1	-18.0 ± 0.03
**20**	44.2 ± 0.1	+0.8± 0.01*	53.8 ± 0.1	+0.31± 0.01*

* S.D. < 0.01; ΔT_m were determined by comparing to the control (1% DMSO)

Table S2. Electron paramagnetic resonance (EPR) parameters assigned to spectra obtained for human phenylalanine hydroxylase incubated with selected 3HQ derivatives.

Compound	Rhombicity (E/D)	Ms	g values
DMSO	**0.327**	**± 3/2**	**4.25**
	0.21	± 1/2	9.20
**3**	**0.327**	**± 3/2**	**4.25**
	0.21	± 1/2	9.20
**9**	**0.33**	**± 3/2**	**4.26**
	0.135	± 1/2	8.5
		± 3/2	5.4
		± 5/2	≈10
	0.04	± 1/2	5.1, 6.8
		± 5/2	≈10
**8**, **11**	**0.33**	**± 3/2**	**4.26**
	0.17	± 3/2	≈3.4, ≈3.1, ≈5.2
		± 5/2	≈10
	0.135	± 1/2	8.5
		± 3/2	5.4
		± 5/2	≈10
	0.04	± 1/2	5.1, 6.8
		± 5/2	≈10

Table S3. Trypsin and Factor Xa (FXa) activity in the presence of 1% DMSO and 100 µM of selected compounds

	Trypsin		Factor Xa	
**Compound**	**ΔAbs/min**	**Residual Activity* (%)**	**ΔAbs/min**	**Residual Activity* (%)**
**1% DMSO**	512 ± 11	100	597 ± 16	100
**3**	403 ± 12	79	N.D.	-
**8**	319 ± 7	62	N.D.	-
**9**	362 ± 9	71	465 ± 29	77
**11**	470 ± 11	92	407 ± 8	69

*Residual Enzyme activity was calculated considering the value obtained for the control (1% DMSO) as 100%; (N.D.) Not determined. Trypsin activity using N-CBZ-GGR-AMC as the enzyme substrate; assays were performed in triplicate. FXa activity using Boc‑Ile‑Glu-Gly‑Arg‑AMC as the enzyme substrate; assays were performed in triplicate.

Table S4. Calculated distances (Å) of the selected 3HQs to the catalytic non-heme iron and its ligands (His285, His290 and Glu330), and to specific amino acid residues that interact with catecholamines reversible inhibitors (Phe254, Tyr325 and Glu330). The compounds were docked into the catalytic centre of hPAH (PDB ID: 3PAH).

Compound	Fe^{2+}- (O)	Phe254	His285	His290	Tyr325	Glu330
**9**	2.15 (OH)<br>2.26 (OC)<br>2.38 (OC)	π-π 3.04	OH-H 2.57	CO-NH 1.9	OC-HO 3.05	CO-H3C 2.14
**11**	1.73(OH)<br>2.25 (OC)	π-π 3.54	CO-H 1.95	CO-NH 2.4	OC-HO 5.19	HO-O 2.66

Table S5. Calculated distances (Å) of the selected 3HQs to specific amino acid residues, involved in cofactor (BH_4) and substrate (L-Phe) binding. The compounds were docked into the catalytic centre of hPAH (PDB ID: 1MMT).

		**9**	**11**
**BH_4**			
	Gly247	C(ar)-(OC) 5.09	C(ar)-(OC) 5.04
	Leu249	O -(HN) 3.39<br>F-(OC) 2.41	O -(HN) 2.84<br>F-(OC) 2.04
	Ser251	F -(OC) 4.85<br>F-(OH) 3.95	F -(OC) 4.92<br>F-(OH) 4.84
	Phe254	C(ar)-C(ar) 2.83	C(ar)-C(ar) 3.10
	Glu286	(CO)-(HO) 1.70	(NH-O) 2.30
L-**Phe**			
	His285	OH-H 2.57	CO-H 1.95
	Ser349	-	-
	Arg270	-	-
	Thr278	-	-
	Tyr128	-	
	Pro281	CO-H2C 3.37<br>HO- H2C 3.08	CO-H2C 3.36<br>HO- H2C 2.35
	Ala 322	-	-
	Trp326	C(ar)-OCH3 2.5, 2.9	C(ar)-OC 3.17
	Phe331	H3C-H 3.3	-
	Ser250	F-(OC) 2.73<br>F-(NH) 3.87	F-(OC) 2.43<br>F-(NH) 3.35
	Gly346	H-CH3 3.20	
	Ser350	-	-

Table S6. Small-angle X-ray scattering data collection and experimental parameters for hPAH.

(*a*) Sample details

	$hPAH^{Buffer}$	$hPAH^{DMSO}$	$hPAH^{Phe}$	$hPAH^{C9}$
Organism	*Homo sapiens*			
Source	*E. coli* Top10			
UniProt sequence ID (residues in construct)	P00439 (1–452)			
Fusion tag	N-terminal hexa-histidine tag (36 additional residues)			
Extinction coefficient ε (280 nm, 0.1% *w*/*v*)	0.910			
Partial specific volume $\bar{\upsilon}$ ($cm^3.g^{-1}$)	0.735			
Mean solute and solvent scattering length densities and mean scattering contrast $\Delta\overline{\rho}$ ($\rho_{protein}$-$\rho_{solvent}$) (10^{10} cm^{-2})	2.585 (12.334-9.475)	2.819 (12.334-9.514)	2.819 (12.334-9.514)	2.819 (12.334-9.515)
Molecular mass *M* from chemical composition (tetramer) (Da)	223 000			
Sample concentration ($mg.mL^{-1}$)	1.0 – 5.0			
Solvent composition	20 mM Hepes, 200 mM NaCl, pH 7.0			
Solvent additives	None	2% DMSO	200 μM L-Phe, 2% DMSO	200 μM **9**, 2% DMSO

(*b*) SAXS data collection parameters

Instrument/data processing	B21, Diamond Light Source (DLS), Harwell, (UK) [30]
Wavelength (Å)	0.95
Collection mode	Static
Beam size (μm)	1102 × 240
Camera length (m)	3.705
s-measurement range ($Å^{-1}$)	0.0031 – 0.380
Absolute scaling method	Comparison with scattering from protein standard (BSA)
Basis for normalization to constant counts	To the integrated intensity from the beam-stop diode
Method for monitoring radiation damage	Frame comparison
Exposure time, number of exposures	1 sec/frame, 31 frames
Sample temperature in sample handling robot (ºC)	10
Sample temperature in flow cell (ºC)	20

(*c*) Software employed for SAXS data reduction

SAXS data reduction	*DAWN* pipeline (DLS, Harwell, UK) [48], ATSAS 3.0.1 [31]
Calculation of ε from sequence	ProtParam [49]
Calculation of $\Delta\overline{\rho}$ and $\bar{\upsilon}$ values from chemical composition	MULCh [50]
Basic analyses: Guinier, *P(r)*, scattering particle volume (V_P)	*PRIMUSqt* [32] from ATSAS 3.0.1

(*d*) Structural parameters

	$hPAH^{Buffer}$	$hPAH^{DMSO}$	$hPAH^{Phe}$	$hPAH^{C9}$
Guinier Analysis				
I(0) (Rel. units)	0.19 ± 0.00009	0.92 ± 0.00120	0.20 ± 0.00014	0.19 ± 0.00017
R_g (Å)	49.43 ± 0.04	50.31 ± 0.71	52.73 ± 0.07	52.14 ± 0.07
s-range ($Å^{-1}$)	0.010 – 0.026	0.012 – 0.025	0.010 – 0.021	0.013 – 0.022
P(r) analysis				
I(0) (Rel. units)	0.19 ± 0.00013	0.92 ± 0.00066	0.19 ± 0.00011	0.19 ± 0.00012
R_g (Å)	51.46 ± 0.06	52.16 ± 0.06	52.37 ± 0.04	52.00 ± 0.05
d_{max} (Å)	193	193	179	189
s-range ($Å^{-1}$)	0.012 – 0.161	0.012 – 0.159	0.011 – 0.156	0.014 – 0.158
χ^2 (total estimate from GNOM)	0.68	0.68	0.61	0.70
Volume (V_P) ($Å^3$)	423 841	427 960	629 301	428 899
M_r from V_P (Da)	265 000	267 000	393 000	268 000

ANNEX

COMPOUND SYNTHESIS AND ANALYSIS

1. General remarks

Dichloromethane (DCM) as reaction solvent was freshly distilled over calcium hydride while absolute ethanol was used without any purification. All reactions were performed in oven-dried glassware. Reaction mixtures were analysed by thin layer chromatography using Merck silica gel 60F254 aluminium plates and visualized by UV light and with phosphomolybdic acid solution.

NMR spectra were recorded in a Bruker Fourier 300 using $CDCl_3$, $(CD_3)_2SO$ as deuterated solvents. All coupling constants are expressed in MHz and chemical shifts (δ) in ppm. Multiplicities are given as: s (singlet), d (doublet), dd (double doublet), dt (double triplet), t (triplet), td (triple triplet), tt (triple triplet), q (quartet), quint (quintuplet) and m (multiplet). Mass spectra were recorded in a mass spectrometer (Micromass Quattro Micro API, Waters, Ireland) with a Triple Quadrupole (TQ) and with an electrospray ion source (ESI) operating in positive or negative mode. Elemental analysis was performed in a Flash 2000 CHNS-O analyser (ThermoScientific, UK).

2. Synthesis of *N*-hydroxysuccinimide (NHS) – Diazoacetate 15

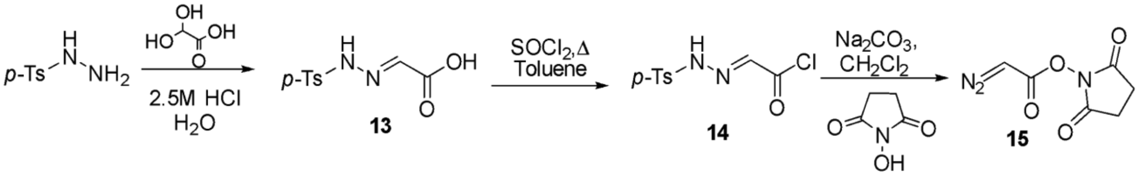

Diazo succinimide **15** was synthesized following literature procedures [1-3] starting from commercially available glyoxylic acid and *p*-toluenesulfonylhydrazine.

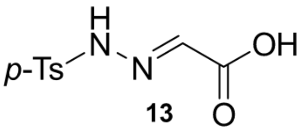

(E)-2-(2-tosylhydrazono)acetic acid 13. 2,2-dihydroxyacetic acid (0.989 g, 10.74 mmol) was dissolved in water (9.9 ml) at 65 °C under magnetic stirring; to this solution, a suspension of *p*-toluenesulfonylhydrazine (2 g, 10.74 mmol) in 2.5 M aqueous HCl (6.01 ml, 15.04 mmol), previously heated to 65 °C, was added at once. The resulting suspension was left under magnetic stirring for 15 minutes at 65 °C, after which it was left to cool down at room temperature and then refrigerated overnight. The resulting crude was filtered over a Büchner funnel and washed with cold water, then dried under vacuum.

The white solid crude was then dissolved in a minimal amount of hot ethyl acetate, then hexane was added dropwise until the solution became cloudy and then boiling ethyl acetate was added dropwise until the solution appeared clear again. The solution was left in the freezer overnight, then the resulting solid was filtered over a Büchner funnel and washed with a cold mixture of hexane:ethyl acetate 2:1. The resulting white solid was dried at high vacuum overnight. No further purification was needed, as the product was obtained as a pure white solid with 70% isolated yield.

^{1}H-NMR spectrum and melting point match the ones described in the literature (Mukherjee, M.; Gupta, A. K.; Lu, Z.; Zhang, Y.; Wulff, W. D. Seeking passe-partout in the catalytic asymmetric aziridination of imines: evolving toward substrate generality for a single chemzyme. *J. Org. Chem.* **2010,** 75, 5643-60)

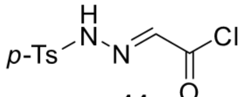

14

(E)-2-(2-tosylhydrazono)acetyl chloride 14. In a flame-dried flask, (E)-2-(2-tosylhydrazono)acetic acid (1.33 g, 5.49 mmol) was suspended in freshly distilled toluene (6.46 ml), then thionyl chloride (0.801 ml, 10.98 mmol) was slowly added dropwise to the flask. The reaction was then heated to reflux under argon atmosphere. The reaction was stopped after 2 hours by quickly cooling the flask to room temperature with a water bath. The reaction solution was filtered through Celite®. Then the filtrate was concentrated at reduced pressure, yielding a yellow solid crude. The crude was dissolved in warm dry toluene, then hexane was added until the formation of precipitate was observed, which was filtered over a Büchner funnel and washed with hexane. No further purification was performed, since the product was obtained as pale yellow crystals, with a yield of 83%. Melting point and ^{1}H-NMR correspond to what is indicated in the literature (Blankley, C. J.; Sauter, F. J.; House, H. O. Crotyl diazoacetate. *Org. Synth.* **1969,** 49, 22).

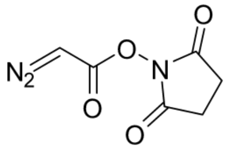

15

NHS – Diazoacetate 15. (E)-2-(2-tosylhydrazono)acetyl chloride (2 g, 7.67 mmol) was dissolved in 20 mL of freshly distilled DCM and slowly added dropwise over 1h to a stirred suspension of *N*-hydroxysuccinimide (0.971 g, 8.44 mmol) and Na_2CO_3 (1.220 g, 11.51 mmol) in 15 mL DCM at 0 °C under argon atmosphere. After the addition was complete, the reaction was kept under stirring at 0 °C for 1 hour and then 3 hours at room temperature, after which the reaction mixture was centrifuged in plastic test tubes. After centrifugation, the supernatant was filtered through Celite® and the filtrate was concentrated at reduced pressure, yielding a pale green solid. The crude was dissolved in a minimal

amount of DCM and then precipitated by dropwise addiction of hexane. The resulting solid was collected and washed with cold hexane, yielding the product as a pale green solid with 43% yield.

The NMR spectra of the product match what is reported in the literature (Doyle, M. P.; Kalinin, A. V. Highly enantioselective intramolecular cyclopropanation reactions of N-allylic-N-methyldiazoacetamides catalyzed by chiral dirhodium(II) carboxamidates. *J. Org. Chem.* **1996,** 61, 2179-2184).

3. Synthesis of 4-NHS-3-hydroxyquinolin-2(1H)-ones 16 and 17

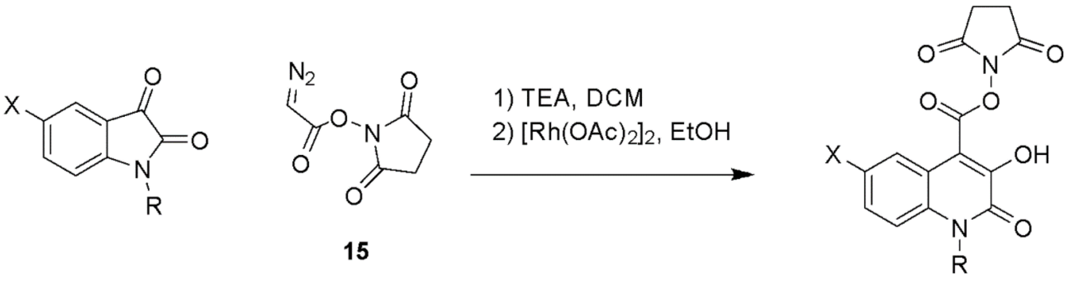

4-NHS-3HQs **16 and 17** were prepared adapting a procedure previously developed in our group (Paterna, R.; Andre, V.; Duarte, M. T.; Veiros, L. F.; Candeias, N. R.; Gois, P. M. P. Ring-Expansion Reaction of Isatins with Ethyl Diazoacetate Catalyzed by Dirhodium(II)/DBU Metal-Organic System: En Route to Viridicatin Alkaloids. *Eur. J. Org. Chem.* **2013,** 2013, 6280-6290).

In a thick-walled vial for centrifuge, 0.606 mmol of the appropriate isatin derivative were dissolved, along with NHS-Diazoacetate (150 mg, 0.818 mmol) in dry DCM (3 ml) and stirred with dry triethylamine (16.88 µl, 0.121 mmol) at room temperature under argon atmosphere. When complete conversion of the starting isatin was observed, the solvent was evaporated at reduced pressure, then the resulting crude was dissolved in absolute ethanol (3 ml), and then diacetoxyrhodium (2.68 mg, 6.06 µmol) was added to the mixture, causing a rapid bubbling of the reaction mixture, with consequent formation of a precipitate. The reaction was stirred for 30 minutes, then it was centrifuged, and the solid was collected by decantation. The resulting precipitate was washed with ethanol and then twice with diethyl ether, yielding the corresponding NHS-3-hydroxyquinolin-2(1H)-one derivatives as pure solids.

Compounds characterization

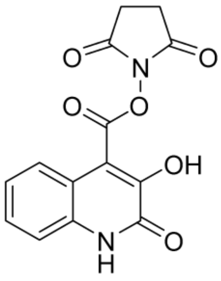

16

4-NHS-3-HQ 16. Grey solid, yield 60%. **^{1}H NMR** (300 MHz, $(CD_3)_2SO$): δ 12.53 (s, 1H), 11.16 (s, 1H), 7.80 (dd, J = 8.1, 1.3 Hz, 1H), 7.46 – 7.33 (m, 2H), 7.26 (ddd, J = 8.3, 6.8, 1.6 Hz, 1H), 2.91 (d, J = 6.4 Hz, 4H). **^{13}C NMR** (75 MHz, $(CD_3)_2SO$): δ 170.72, 161.64, 157.68, 147.46, 133.31, 127.90, 123.72, 123.48, 116.98, 116.14, 111.04, 26.12. **LRMS (m/z)**: calculated for $[M+H^+]$ $C_{14}H_{11}N_2O_6$: 303.1, found: 303.2.

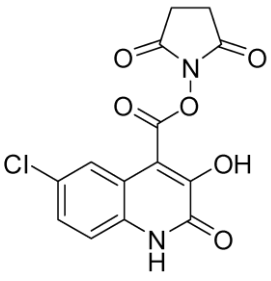

17

4-NHS-3-HQ 17. Grey solid, yield 50%. **^{1}H NMR** (300 MHz, $(CD_3)_2SO$): δ 12.59 (s, 1H), 7.92 (d, J = 2.1 Hz, 1H), 7.43 (d, J = 2.2 Hz, 1H), 7.35 (d, J = 8.7, 1H), 2.92 (s, 4H). **^{13}C NMR** (75 MHz, $(CD_3)_2SO$): δ 170.34, 160.85, 157.25, 149.27, 131.46, 127.13, 126.96, 122.44, 118.32, 117.48, 108.77, 25.69. **LRMS (m/z)**: calculated for $[M+H^+]$ $C_{14}H_{10}ClN_2O_6$: 337.0, found: 337.3.

4. Synthesis of Phe-3HQs 1 and 2

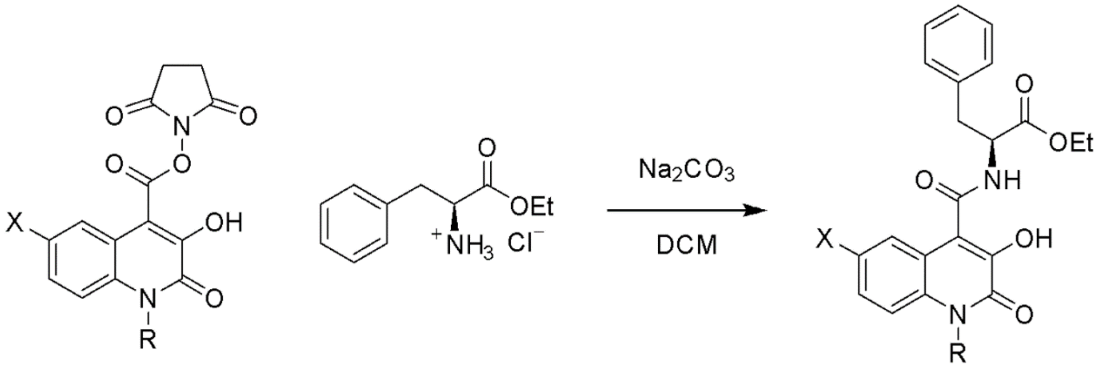

1: X=H, R=H
2: X=Cl, R=H

Phenylalanine ethyl ester hydrochloride (50 mg, 0.22 mmol, 1.1 eq) and Na_2CO_3 (212 mg, 2 mmol, 10 eq) were added to a stirred solution of the appropriate 4-NHS-3HQ (0.2 mmol) in dry DCM (1 mL). The mixture was stirred overnight at room temperature after which the volatiles were evaporated under reduced pressure and the crude mixture acidified with HCl (2 N). The precipitate was then centrifuged and collected by filtration. Finally, the isolated solid was thoroughly washed with H_2O to furnish the desired Phe-3HQs.

Compounds characterization

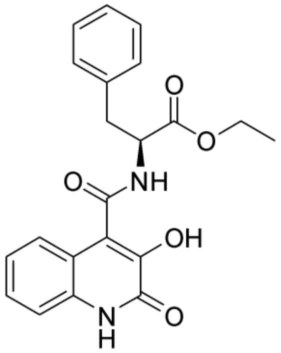

1

Phe-3HQ 1. Grey solid, yield 93%. **^{1}H NMR** (300 MHz, $(CD_3)_2SO$): δ 12.03 (s, 1H), 9.71 (s, 1H), 7.60 – 6.82 (m, 9H), 4.86 – 4.50 (m, 2H), 4.09 (q, J = 7.1 Hz, 2H), 3.04 (ddd, J = 22.8, 13.9, 7.6 Hz, 1H), 1.15 (t, J = 7.1 Hz, 3H). **^{13}C NMR** (75 MHz, $(CD_3)_2SO$): δ 171.66, 165.56, 160.02, 142.03, 137.36, 132.34, 129.37, 128.38, 126.62, 125.66, 124.12, 123.31, 122.08, 121.71, 119.50, 114.94, 60.64, 54.00, 14.08.

LRMS (m/z): calculated for [M+Na$^+$] $C_{21}H_{20}N_2O_5Na$: 403.1, found: 403.7. **Elemental analysis** calculated (%) for $C_{21}H_{20}N_2O_5$ + 0.5 H_2O: C, 61.10; H, 5.76; N, 6.79; Found: C, 60.87; H, 5.56; N, 6.65.

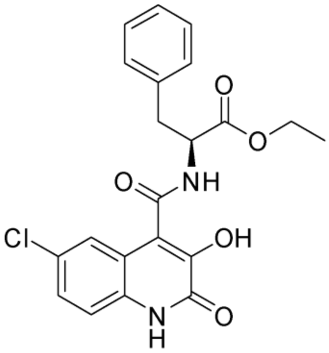

2

Phe-3HQ 2. Grey solid, yield 87%. **^{1}H NMR** (300 MHz, $(CD_3)_2SO$): δ 12.31 (s, 1H), 9.10 (s, 1H), 7.46 – 7.10 (m, 8H), 4.63 (dd, J= 14.2, 8.0 Hz, 2H), 4.12 (q, J = 7.0 Hz, 2H), 3.04 (ddd, J = 23.1, 13.9, 7.6 Hz, 1H), 1.17 (t, J = 7.1 Hz, 3H).**^{13}C NMR** (75 MHz, $(CD_3)_2SO$): δ 171.60, 164.51, 164.47, 158.42, 143.73, 137.49, 132.07, 129.54, 128.60, 126.98, 126.84, 126.73, 123.22, 120.18, 119.57, 117.28, 61.06, 54.44, 14.36 **LRMS (m/z)**: calculated for [M+Na$^+$] $C_{21}H_{19}N_2O_5ClNa$: 436.4, found: 436.6. **Elemental analysis** calculated (%) for $C_{21}H_{19}N_2O_5Cl$ + 1.1 H_2O: C, 58.03; H, 4.92; N, 6.45; Found: C, 58.24; H, 4.87; N, 6.25.
